# Gene expression analysis of potential morphogen signalling modifying factors in Panarthropoda

**DOI:** 10.1186/s13227-018-0109-y

**Published:** 2018-09-29

**Authors:** Mattias Hogvall, Graham E. Budd, Ralf Janssen

**Affiliations:** Department of Earth Sciences, Palaeobiology, Uppsala University, Villavägen 16, Uppsala, Sweden

**Keywords:** Gene regulatory networks, Development, Evolution, Arthropoda, Panarthropoda, Onychophora

## Abstract

**Background:**

Morphogen signalling represents a key mechanism of developmental processes during animal development. Previously, several evolutionary conserved morphogen signalling pathways have been identified, and their players such as the morphogen receptors, morphogen modulating factors (MMFs) and the morphogens themselves have been studied. MMFs are factors that regulate morphogen distribution and activity. The interactions of MMFs with different morphogen signalling pathways such as Wnt signalling, Hedgehog (Hh) signalling and Decapentaplegic (Dpp) signalling are complex because some of the MMFs have been shown to interact with more than one signalling pathway, and depending on genetic context, to have different, biphasic or even opposing function. This complicates the interpretation of expression data and functional data of MMFs and may be one reason why data on MMFs in other arthropods than *Drosophila* are scarce or totally lacking.

**Results:**

As a first step to a better understanding of the potential roles of MMFs in arthropod development, we investigate here the embryonic expression patterns of *division abnormally delayed* (*dally*), *dally-like protein* (*dlp*), *shifted* (*shf*) and *secreted frizzled-related protein 125* (*sFRP125*) and *sFRP34* in the beetle *Tribolium castaneum*, the spider *Parasteatoda tepidariorum*, the millipede *Glomeris marginata* and the onychophoran *Euperipatoides kanangrensis*. This pioneer study represents the first comprehensive comparative data set of these genes in panarthropods.

**Conclusions:**

Expression profiles reveal a high degree of diversity, suggesting that MMFs may represent highly evolvable nodes in otherwise conserved gene regulatory networks. Conserved aspects of MMF expression, however, appear to concern function in segmentation and limb development, two of the key topics of evolutionary developmental research.

**Electronic supplementary material:**

The online version of this article (10.1186/s13227-018-0109-y) contains supplementary material, which is available to authorized users.

## Background

The successful development of an animal largely relates to the so-called organizing centres (OCs). An OCs is a region within the developing embryo that produces one or more secreted signalling molecules that provide positional information to its “nearby” cellular environment.

The combination of multiple OCs establishes defined patterns of gene activity and thus of differentiating cell types within an embryo. One critical challenge for OCs and their signalling molecules is the proper coordination of their action in space and time. If this fine-tuning fails, this usually leads to abnormal and fatal development.

Important families of signalling molecules are represented by the Hedgehog (Hh) family of genes, the Wnt genes and bone morphogenic proteins (BMPs) such as Decapentaplegic (Dpp). The products of these genes act as morphogens since they form gradients that cause cells along these gradients to develop into different identities (reviewed in [1–6]). Although it is possible that morphogen gradients form by the mere diffusion through the extracellular space, it appears much more likely that the formation of these gradients relies on other factors that are either connected to the cell surface or that are diffusing through the extracellular space (reviewed in [7–10]). Indeed, control of regulation of morphogens and their co-factors is of the uttermost importance for the developing organism. Therefore, regulation occurs on several levels: for example, transcriptional control at the sources of morphogen production (these are the OCs) or control of morphogen transport to their respective target cells [11–18].

Altogether, these data reveal a high level of complexity that underlies morphogen activity in developing animal embryos. Our knowledge, however, is often restricted to data from model organisms such as the vinegar fly *Drosophila melanogaster*, by far the best understood arthropod model organism. Although *Drosophila* development has been studied in great detail, it is still unclear how exactly morphogen gradients form, even in this species, and how their distribution is controlled in the extracellular space (e.g. [19]). When it comes to other arthropods, or panarthropods, our knowledge is even more scarce, and most studies only address the key players of morphogen signalling pathways, i.e. their regulatory activators (transcription factors), the morphogens themselves, and their receptors (e.g. [20–24]). To our knowledge, there are no data on factors that may be involved in morphogen trafficking and regulation after their secretion into the extracellular space in panarthropod species other than *Drosophila*, except for some gene expression data on the secreted extracellular hydrolase *Notum* (aka *Wingful*) in a spider, a myriapod and an onychophoran [23, 25, 26].

In order to provide a basis for understanding how morphogen gradients are regulated in arthropods and their closest relatives, the onychophorans, we studied gene expression patterns of genes that are likely involved in this process. We have chosen representatives of all main lineages of arthropods (Pancrustacea, Myriapoda, Chelicerata) and a panarthropod species (Onychophora) to gain comprehensive insight into potentially conserved and derived mechanisms of morphogen regulation. In previous studies, we (and others) investigated the mRNA distribution of Wnt genes [21, 23, 24, 26, 27–36], Hedgehog (Hh) orthologs [22, 23, 26, 28, 37, 38], their Frizzled receptors [34, 39] and Patched [22, 23, 26, 37], Decapentaplegic (Dpp) [27, 30, 40–42] and the hydrolase Notum [23, 25, 26] in these panarthropods.

Here, we extend this analysis to a number of genes that are known to interact with morphogen signalling. In detail, we investigated the embryonic expression patterns of the glypican encoding genes *division abnormally delayed* (*dally*) and *dally*-*like protein* (*dlp*), *shifted* (*shf*) (aka *Wnt Inhibitory Factor 1* (*WIF1*)), and the *secreted Frizzled*-*related protein125* (*sFRP125*) and *sFRP34*. We name these genes “morphogen signalling modulating factors (MMFs)”. Numerous previous studies have shown that these genes are involved in morphogen signalling in various different animal groups [14, 15, 43–47].

We compare our new data with the previously published differential expression patterns of genes involved in morphogen signalling. With respect to evolutionary conserved patterns, we focus in the present study on morphogen signalling in limb development and AP body axis patterning (i.e. body segmentation), two of the key topics in panarthropod evolutionary developmental research (e.g. [48–55]). However, most of the MMFs are expressed in diverse patterns suggesting that they may represent a group of genetic factors that have been free to evolve and thus may contribute to species- and clade-specific morphological features.

## Methods

### Embryos and developmental staging

Embryos of the red flour beetle *Tribolium castaneum*, the common pill millipede *Glomeris marginata*, the cosmopolitan house spider *Parasteatoda tepidariorum* and the velvet worm *Euperipatoides kanangrensis* were obtained as described in Grossmann and Prpic [56] (*Tribolium*), Janssen et al. [28] (*Glomeris*), Prpic et al. [57] (*Parasteatoda*) and Hogvall et al. [24] (*Euperipatoides*). Developmental staging is after Janssen et al. [28] (*Glomeris*), Janssen and Budd [26] (*Euperipatoides*), Mittmann and Wolff [58] (*Parasteatoda*), and Strobl and Stelzer [59] (*Tribolium*).

### Gene cloning, whole-mount in situ hybridization and nuclear staining

Gene fragments were amplified by means of RT-PCR from cDNA synthesized from either total RNA or messenger RNA. Gene-specific primers were designed based on available sequence information (Tribolium Genome Sequencing Consortium [26, 35, 60, 61] (Additional file 1: Table S1)).

All amplified gene fragments were cloned into the PCRII vector (Invitrogen). Sequences of the cloned fragments were sequenced on an ABI3730XL automatic sequencer (Macrogen, Seoul, South Korea). Gene fragment identification numbers are summarized in Additional file 2: Table S2.

The whole-mount in situ hybridization protocol was used as described in [62]; for confocal microscopy, we stained embryos with SIGMAFAST Fast Red TR/Naphtol AS-MX (SIGMA) instead of BM Purple (ROCHE). Cell nuclei were visualized incubating embryos in 5 μg/ml of the fluorescent dye 4-6-diamidino-2-phenylindole (DAPI) in phosphate buffered saline with 0.1% Tween-20 (PBST) for 20 min, followed by several washes in PBST to remove excess DAPI.

### Phylogenetic analysis

Amino acid sequences of Smoothened (Smo), Secreted Frizzled-Related Proteins 125 and 34, Netrin (Analysis 1), and Dally and Dally-like proteins (Analysis 2) were aligned using ClustalX with default parameters in MacVector v12.6.0 (MacVector, Inc., Cary, NC). In Analysis 1, the *Drosophila* Frizzled-2 (Fz2) gene serves as outgroup. Structurally-related Netrin and Smoothened protein sequences have been added to ensure that neither of our investigated genes represent *netrin*. In Analysis 2, a glypican from the demosponge *Amphimedon* serves as an outgroup. Bayesian phylogenetic analyses were performed with MrBayes [63] using a fixed WAG amino acid substitution model with gamma-distributed rate variation across sites (with four rate categories). Unconstrained exponential prior probability distribution on branch lengths and an exponential prior for the gamma shape parameter for among-site rate variation were applied. Topologies were in each case estimated using 1,000,000 cycles for the MCMCMC (metropolis-coupled Markov chain Monte Carlo) analysis with four chains and the chain-heating temperature set to 0.2. The Markov chains were sampled every 200 cycles. We used default settings of 25% of samples as burnin. Clade supports were calculated with posterior probabilities computed with MrBayes.

### Data documentation

Embryos were photographed using a Leica DC100 digital camera mounted onto a Leica dissection microscope. For confocal microscopy, we used an inverted Leica TCS SP5 confocal microscope. When appropriate, brightness and contrast were modified using the image-processing software Adobe Photoshop CC for Apple Macintosh (Adobe Systems Inc.). Embryos of *Tribolium castaneum* were incubated in 87% glycerol, and yolk was removed using fine tungsten needles (recycled from old light bulbs). These embryos were then mounted on glass slides under a thin glass cover. Prior to the dissection of limbs of *Parasteatoda*, embryos were incubated in 87% glycerol. Limb preparations were carried out with ultra-fine tungsten needles sharpened in the flame of a Bunsen burner.

## Results

### Sequence analysis

**Fig. 1:**
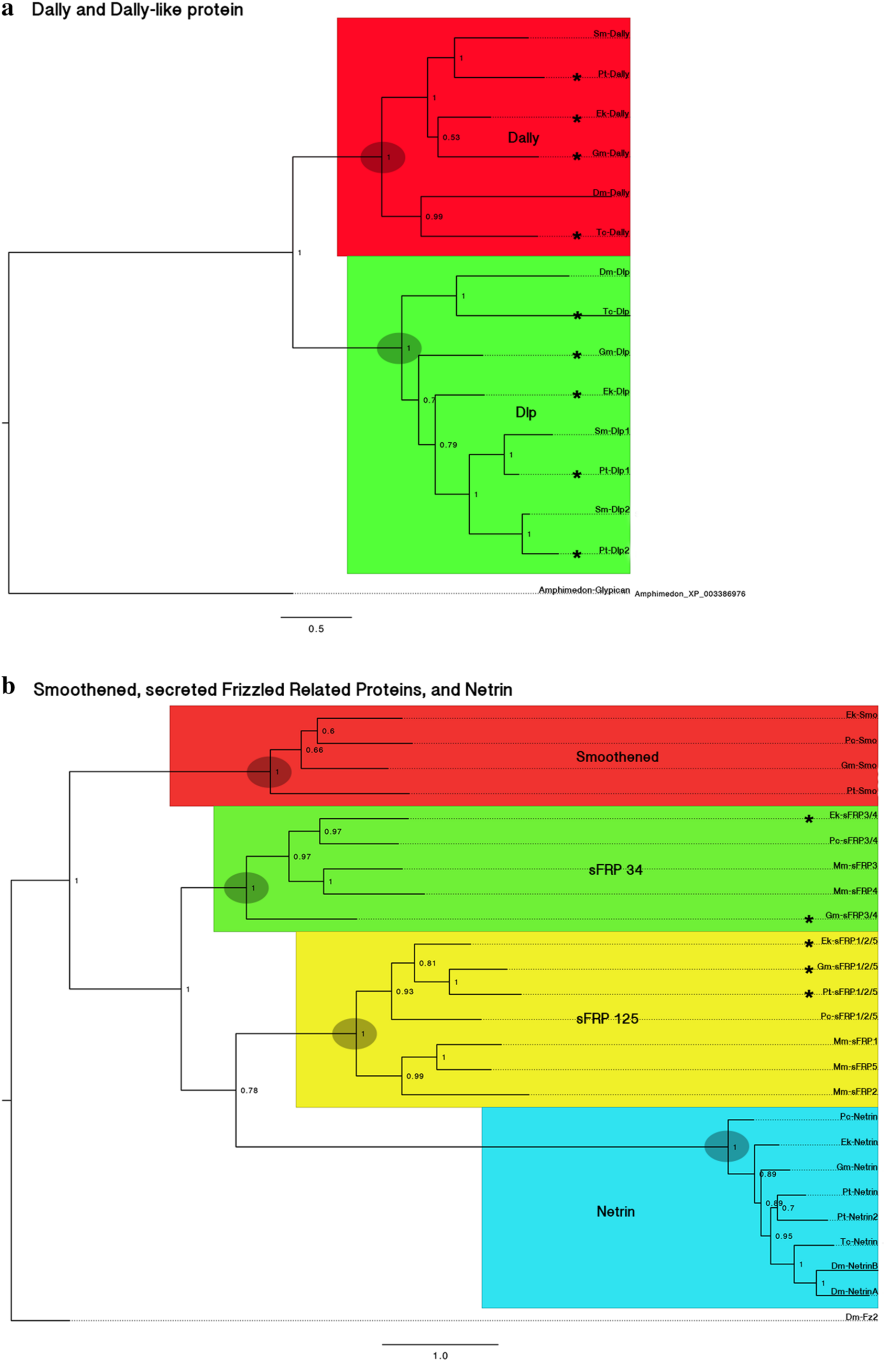
Bayesian inference analysis showing the distribution of panarthropod Dally and Dlp (**a**) and Smoothened, secreted Frizzled-Related Proteins, and Netrin (**b**). Genes investigated in this study are highlighted with asterisks (*). Posterior probabilities > 0.5 are indicated. See text for further information. Species abbreviations: Dm, *Drosophila melanogaster* (Hexapoda: Diptera); Ek, *Euperipatoides kanangrensis* (Onychophora); Gm, *Glomeris marginata* (Myriapoda: Diplopoda); Mm, *Mus musculus* (Vertebrata); Pc, *Priapulus caudatus* (Priapulida); Pt, *Parasteatoda tepidariorum* (Chelicerata: Araneae); Sm, *Stegodyphus mimosarum* (Chelicerata: Araneae); Tc, *Tribolium castaneum* (Hexapoda: Coleoptera)

Our first phylogenetic analysis shows that Dally and Dally-like protein (Dlp) encoding genes cluster separately with unambiguous support (Fig. 1a). It further reveals that there is a single Dally ortholog in each investigated species, but two paralogs of Dlp in each spider species. The latter is likely due to a whole genome duplication in the lineage leading to spiders [61]. Our second phylogenetic analysis shows that predicted Smoothened proteins, Secreted Frizzled-like proteins 125, Secreted Frizzled-like Proteins 34 and Netrins each form monophyletic groups with high support (Fig. 1b).

### Expression of *division abnormally delayed* (*dally*)

**Fig. 2:**
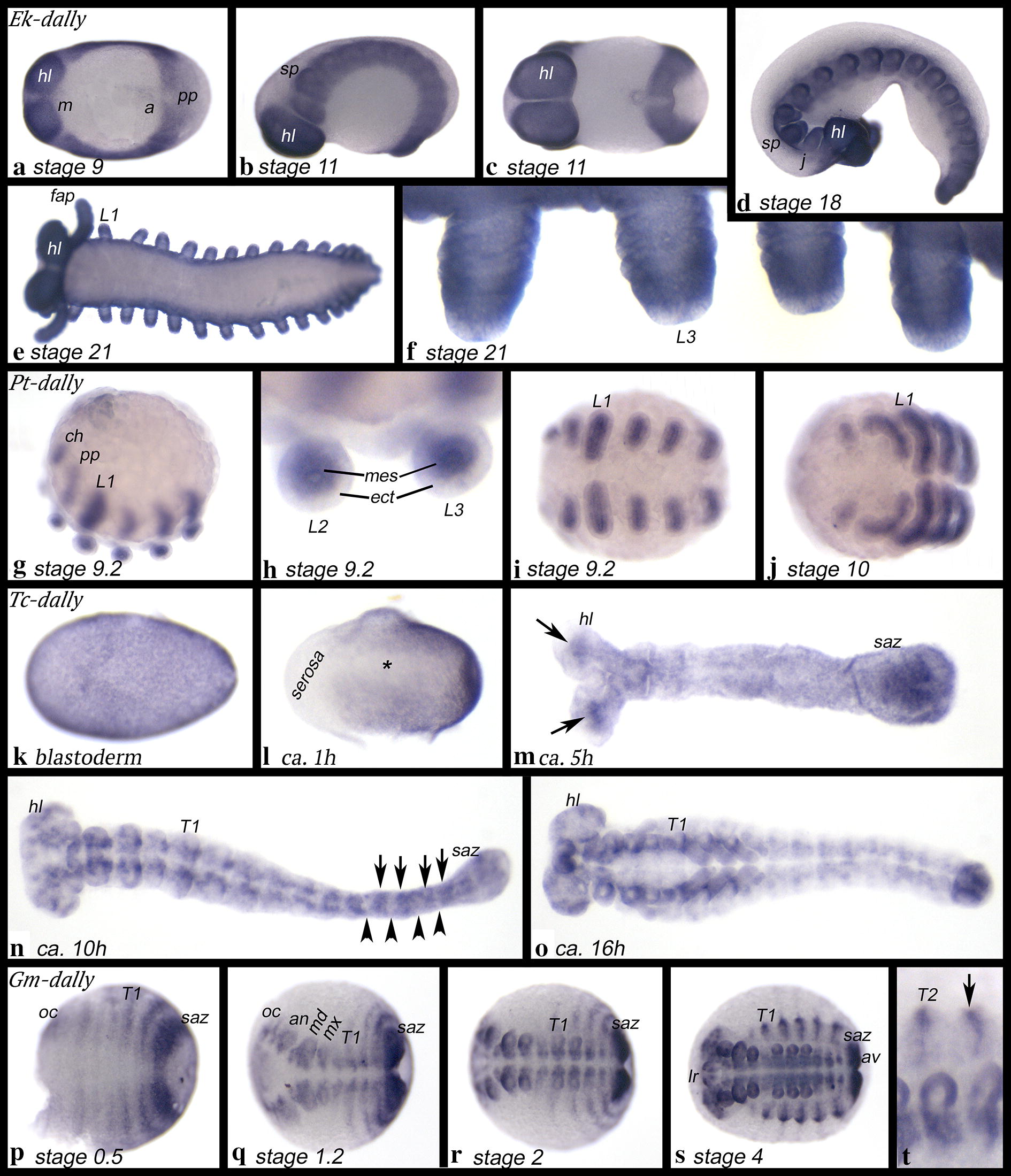
Expression of *dally* in *Euperipatoides* (**a**–**f**), *Parasteatoda* (**g**–**j**), *Tribolium* (**k**–**o**) and *Glomeris* (**p**–**t**). In all panels, anterior is to the left. Ventral views, except for **b**, **d** and **g** (lateral views). The asterisk (*) in **l** mark future mesoderm. Arrows in **m** point to expression in the head lobes. Arrows in **n** point to regions of expression, while arrowheads point to regions of no expression. The arrow in **t** points to a dorsal transverse stripe of expression. a, anus; an, antenna; ch, chelicera; av, anal valve; ect, ecdoderm; fap, frontal appendage; hl, head lobe; j, jaw; L, walking leg; lr, labrum; mes, mesoderm; m, mouth; md, mandible; mx, maxilla; oc, ocular region; pp, pedipalp; saz, segment addition zone; sp, slime papilla; T, trunk segment

Expression of *Euperipatoides dally* (*Ek*-*dally*) is ubiquitous, but the level of expression is much lower in the tips of the appendages (Fig. 2a–f).

*Parasteatoda dally* (*Pt*-*dally*) is expressed in the mesoderm of all limbs and a small anterior and ventral sector of the limb-ectoderm (Figs. 2g–j, 8g–l).

*Tribolium dally* (*Tc*-*dally*) is expressed ubiquitously at the early blastoderm stage (Fig. 2k). With the formation of the germ band, the most ventral region of the embryo (the forming mesoderm) and the most anterior region of the embryo (the serosa) remain free from expression (Fig. 2l). During germ band formation, *Tc*-*dally* is expressed ubiquitously (not shown), but approximately 5 h after gastrulation *Tc*-*dally* becomes expressed differentially in the head; the most anterior region does not express *dally*, while a strong domain of expression appears in the centre of each head lobe (Fig. 2m). Later, expression is in a complex pattern in the head lobes and along the anterior–posterior body axis on either side of the ventral midline; this expression is likely associated with the ventral nervous system (Fig. 2n, o). In the legs, *dally* is strongly expressed at the base and in the form of a sub-terminal ring, while the tips of the legs do not express *dally* (Fig. 2o).

*Glomeris dally* (*Gm*-*dally*) is initially expressed in broad transverse stripes in the *regio germinalis* (the part of the embryo that develops from the blastoderm), in the segment addition zone (SAZ), and in newly-formed posterior segments (Fig. 2p, q). At later stages, expression in the segments becomes largely restricted to the ventral nervous system and the limbs (Fig. 2q–t). In dorsal segmental units, segmental expression persists in the form of short transverse stripes that are likely associated with the formation of the tergite boundaries (e.g. [28]). Double in situ staining with the segmental marker *engrailed* (*en*) which is expressed in the posterior of each segment [23, 28] shows that the stripes of *dally* expression are broader extending further anterior than those of *en*. Tissue posterior of *en* does not express *dally*. It is unclear if *dally* and *en* are co-expressed in ventral tissue (Additional file 3: Figure S1). In dorsal tissue, *en* and *dally* are co-expressed in the form of transverse segmental stripes as the expression of *en *+ *dally* is not broader than the expression of either *en* or *dally* (cf. Figs. 2s, t and S1B with [28]).

### Expression of *dally-like protein* (*dlp*)

**Fig. 3:**
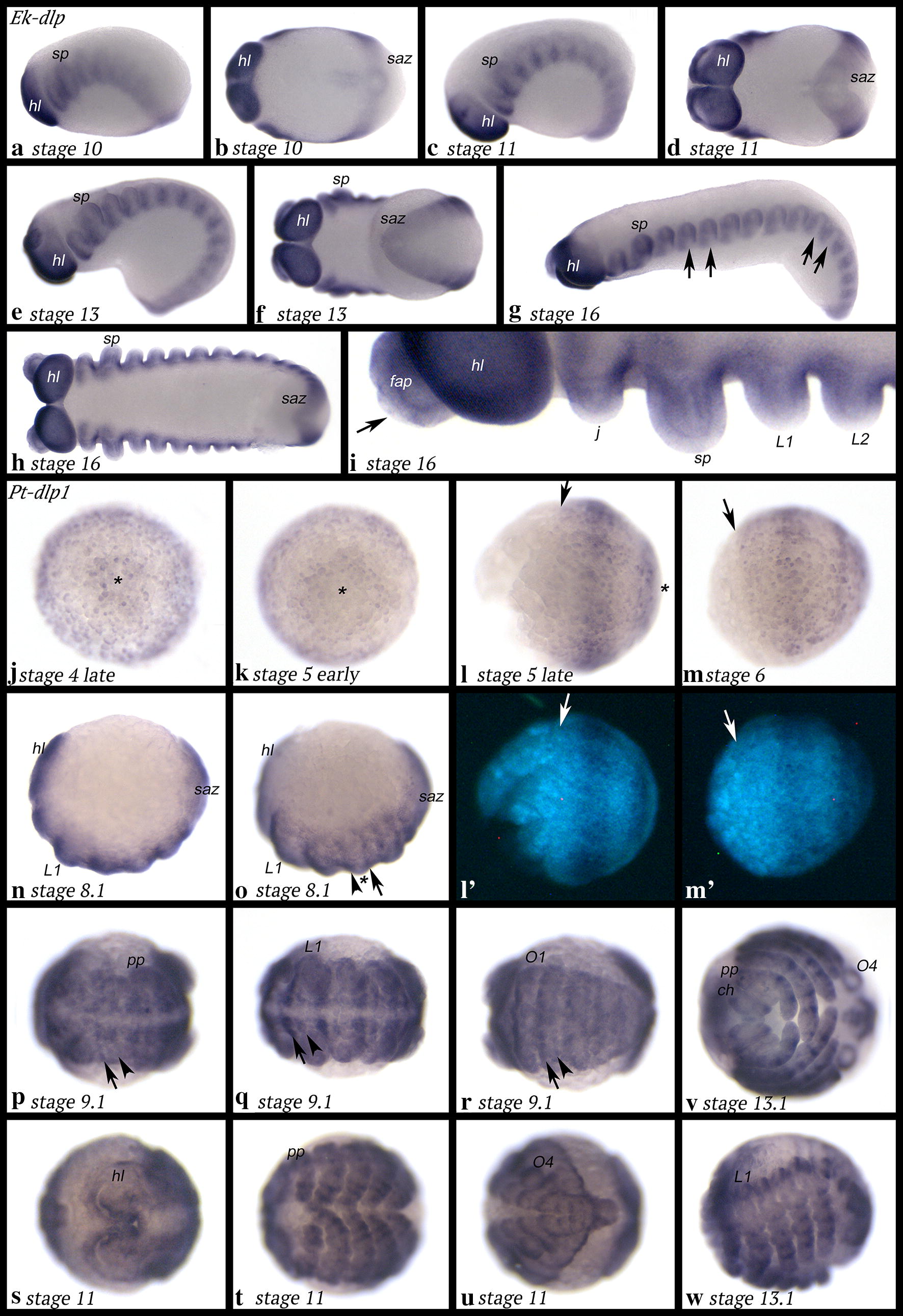
Expression of *dlp* in *Euperipatoides* (**a**–**i**) and *Parasteatoda* (**j**–**w**). In all panels, anterior is to the left. Ventral views, except for panels **a**, **c**, **e**, **g**, **n**, **o** and **w** (lateral views). **l**′, **m**′ Represent DAPI stained embryos as seen in **l**, **m**. Arrows in **g** point to regions of no expression. The arrow in **i** points to lack of expression in the tip of a frontal appendage. Asterisks (*) in **j**–**l** mark the centre of the germ disc. Arrows in **l** and **m** point to the anterior edge of the germ disc. Arrows in **o**-**r** point to stronger stripe-expression, arrowheads in the same panels point to weaker stripe-expression. The asterisk in **o** marks the middle of a limb bud with weaker expression. Abbreviations as in Fig. 2; O, opisthosomal segment

*Euperipatoides dally*-*like protein* (*Ek*-*dlp*) is ubiquitously expressed at early developmental stages (not shown). Later, expression either disappears (or becomes much weaker) from tissue in the central region of the limb buds and corresponding segmental tissue or is upregulated in tissue flanking this region; we cannot distinguish between these two possibilities (Fig. 3a, c, e, g–i). The tips of the appendages do not express *Ek*-*dlp* (Fig. 3h, i). Expression in the SAZ is weaker than in more anterior tissue throughout segment addition (Fig. 3b, d, f).

*Parasteatoda dally*-*like protein 1* (*Pt*-*dlp1*) is expressed at the germ disc stage in the form of two domains, a broad peripheral ring (but note that the most outermost cells of the disc do not express *Pt*-*dlp1*), and a central domain (Fig. 3j). At a slightly later stage, this latter domain transforms into a second broad ring (Fig. 3k–m). With the formation of the germ band and the beginning of limb development, it becomes clear that *Pt*-*dlp1* is expressed at lower levels in the middle of each limb bud (Fig. 3n, o). This results in two stripes per segment, one of which is more pronounced than the other (Fig. 3p–r). Later, when the limbs have further developed, stripes of expression appear in all appendages (Figs. 3s–w, 8m–r). These stripes are restricted to dorsal tissue. In the pedipalps and the chelicerae, there is weak ubiquitous expression of *dlp1* in ventral tissue (Fig. 8m–r). Additional expression is in the most dorsal tissue of the embryo (Fig. 3u); this expression is possibly associated with the formation of the dorsal tube (= heart) (cf. [64]).

**Fig. 4:**
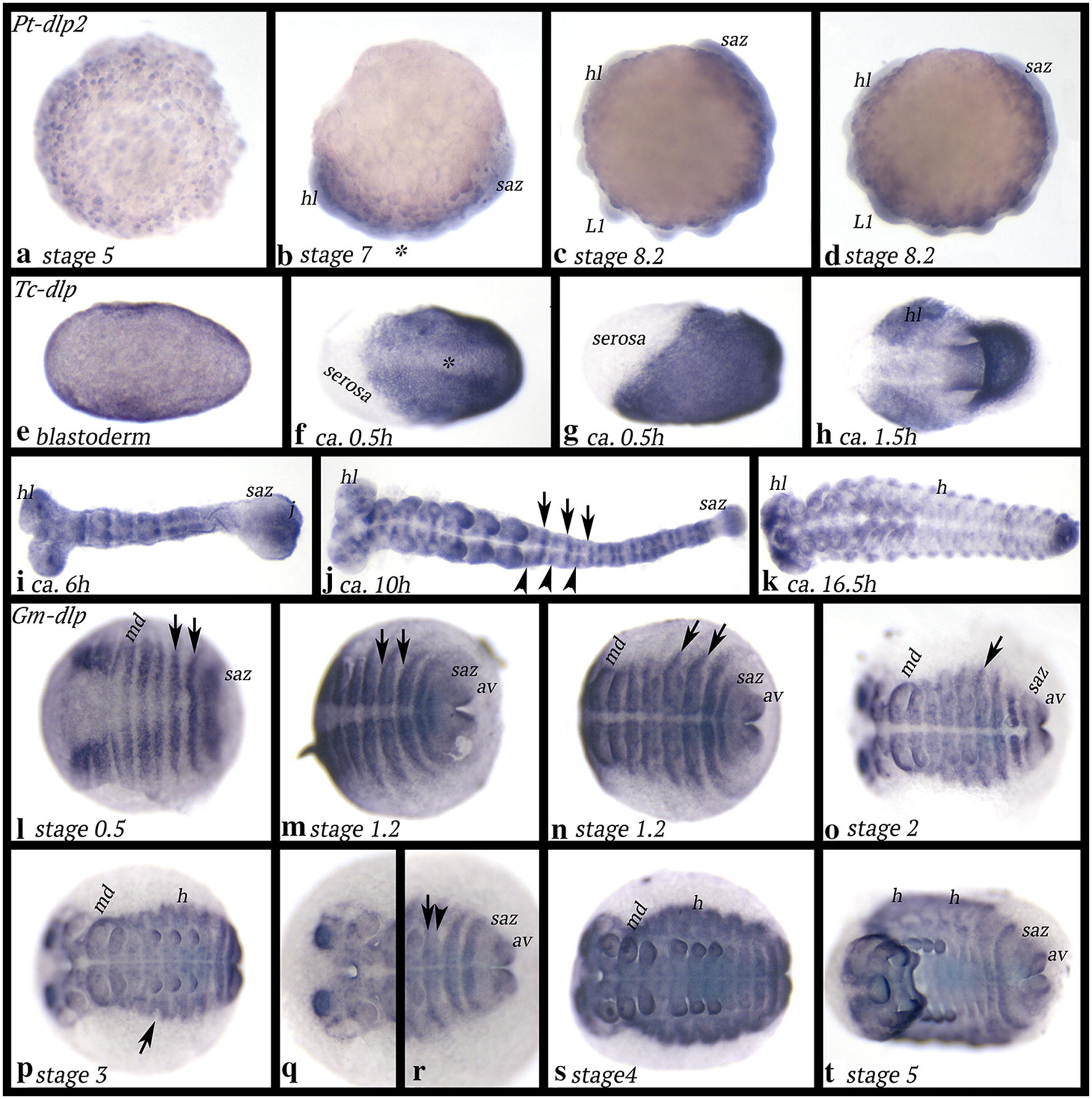
Expression of *dlp* in *Parasteatoda* (*dlp2*) (**a**–**d**), *Tribolium* (**i**–**k**) and *Glomeris* (**l**–**t**). In all panels, anterior is to the left. Ventral views, except for **b**–**d**, and **g** (lateral views). The asterisk in **b** marks region without expression. The asterisk in **f** marks future mesoderm. Arrows and arrowheads in **j** point to stronger and weaker stripes of expression, respectively. Arrows in **l**–**o** point to stripe-expression. Arrow in **p** points to the middle of a dorsal segmental unit that does not express *dlp*. Arrow and arrowhead in **r** point to a stronger anterior and a weaker posterior stripe of expression, respectively. Abbreviations as in Fig. 2; h, heart

*Pt*-*dlp2* is expressed similarly to that of *dlp1* at the germ disc stage (Fig. 4a). With beginning of germ band elongation, *dlp2* is expressed in all tissue except for a gap of expression in the middle of the young germ band (Fig. 4b). Likely, this gap is in the first or second walking leg bearing segment (or the space in between) (cf. [58]). Later, expression disappears from the space between the limb buds in the anterior embryo resulting in a transient segmental pattern of expression; at the same time, expression in the posterior of the embryo, the SAZ, is still ubiquitous (Fig. 4c, d). In all later stage embryos, *dlp2* is expressed ubiquitously (not shown).

*Tribolium dally*-*like protein* (*Tc*-*dlp*) is ubiquitously expressed at the blastoderm stage (Fig. 4e). *Tc*-*dlp* is not expressed in the forming serosa (Fig. 4f, g). Expression along the ventral midline, in the presumptive mesoderm, is weaker than in the rest of the now forming germ band. Stronger expression is seen in the early head lobes (Fig. 4h), and with the beginning of germ band elongation, this expression in the head becomes refined to smaller domains. At the same time, segmental stripes of *dlp* expression appear. Likely, there are two transverse stripes of expression per segment, a stronger anterior stripe and a weaker posterior stripe (Fig. 4i). This becomes even more prominent as more segments are added from the posterior SAZ (Fig. 4j). While there are nine or ten abdominal segments formed in the embryo shown in panel j, there are 18 or 19 stripes of expression. The intra-segmental position of the stripes and segmental correlation are not clear from the currently available data. However, using the limb buds in the head and the thorax as morphological markers, it appears that one stripe is level with the centre of the limb buds, i.e. likely co-expressed with *wingless/Wnt1*. The other stripe is likely anterior in each segment. The SAZ expresses *dlp*, but the tissue just in front of the SAZ is free of expression (Fig. 4j). With the end of germ band elongation and the beginning of germ band retraction, the transverse stripes of expression become weaker (Fig. 4k), but expression in the head lobes (brain) is stronger, and dorsal patches of *dlp*-expressing cells appear; this latter expression is possibly associated with the development of the dorsal tube (= heart) (cf. [64]). In the developing appendages, *dlp* is upregulated in the form of segmental patches (or stripes/rings). Expression of the rings is stronger in dorsal tissue (Additional file 4: Figure S2). Note that this expression is very similar to that of *Pt*-*dlp1*.

*Glomeris dally*-*like protein* (*Gm*-*dlp*) is expressed in broad transverse segmental stripes in newly forming segments (Fig. 4l–p, r–t). The most posterior of the embryo, the posterior of the anal valves, also expresses *dlp*, but the SAZ is free from expression (Fig. 4l–p, r–t). The ventral midline does not express *dlp* (Fig. 4m–p). In the dorsal segmental units, expression first forms as one stripe (Fig. 4n, o), but later a second stripe appears per unit leaving the centre of each unit free from transcription (Fig. 4p, r). At late developmental stages, the most dorsal tissue expresses *dlp*, except for the last two formed segments and the SAZ (Fig. 4t). This expression is possibly associated with the formation of the heart (= dorsal tube) (cf. expression of the heart marker *H15.1* [65]).

### Expression of *secreted frizzled-related protein 125* (*sFRP125*)

**Fig. 5:**
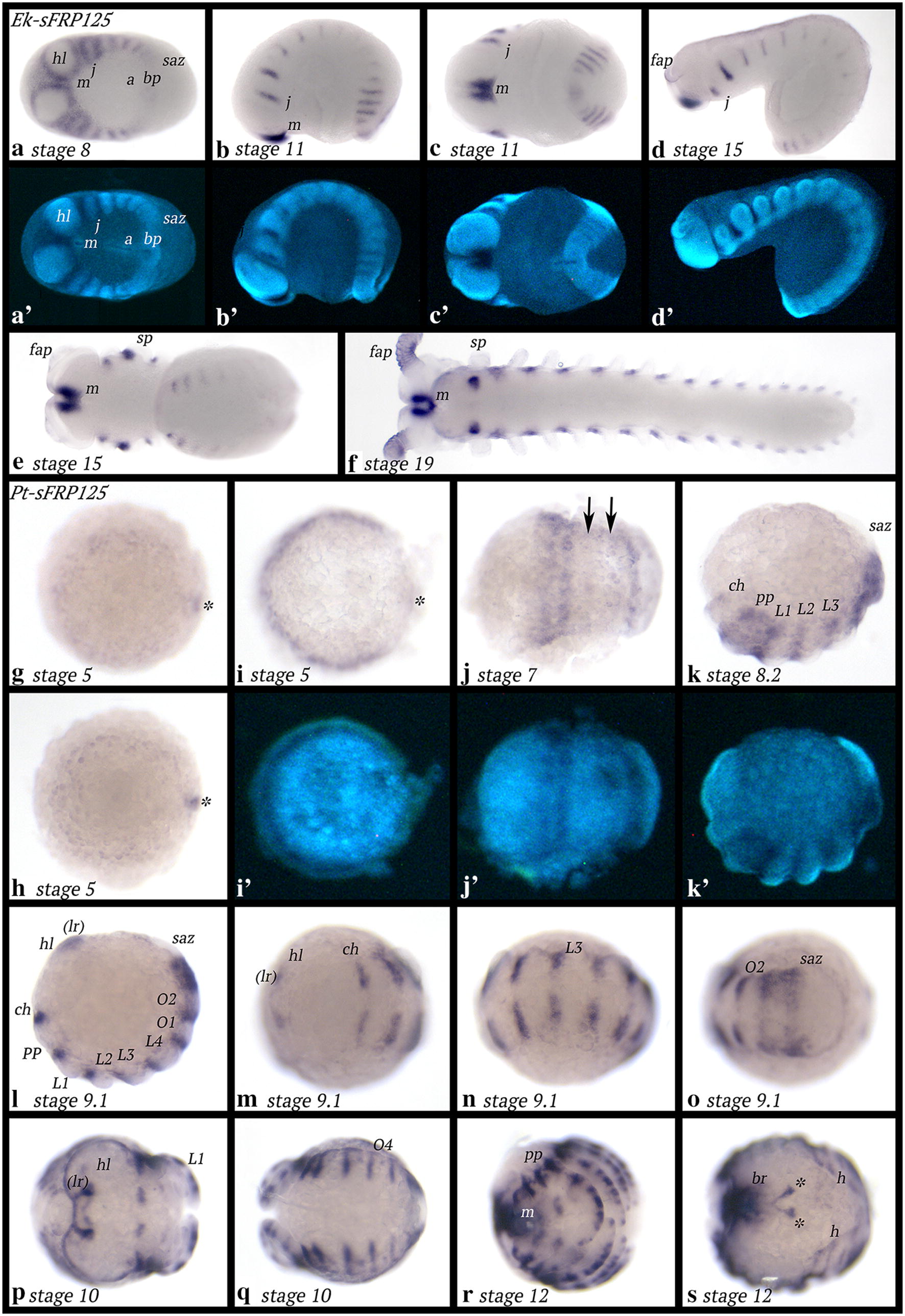
Expression of *sFRP125* in *Euperipatoides* (**a**–**f**) and *Parasteatoda* (**g**–**s**). In all panels, anterior is to the left. Ventral views, except for **b**, **d**, **k** and **l** (lateral views); **r**, (anterior view); **s** (dorsal view). **a**′–**d**′, **i**′–**k**′ Represent DAPI-stained embryos as seen in (**a**–**d**, **i**–**k**). Asterisks in **g**, **i**, **h** point to expression in the cumulus. Asterisks in **s** mark expression anterior to the head lobe contributing to the future heart Abbreviations as in Fig. 2; (lr), region where the labrum will form; bp, blastopore; br, brain; h, heart

In early developmental stages, *Euperipatoides sFRP125* is expressed in all ectodermal anterior tissue, except for the centre of the segments (Fig. 5a). The posterior pit region (= blastopore) expresses *sFRP125* weakly; the mouth-anus furrow and the SAZ do not express *sFRP125* (Fig. 5a). Later, the expression pattern transforms into segmental transverse stripes that are located between the limb buds, that now start to grow out (Fig. 5b). Interestingly, although the stripes in the anterior of the embryo and the posterior of the embryo appear similar, they are separated by segments in the middle of the body, that either do not or only weakly express *sFRP125* (Fig. 5b, d). Confocal microscopy reveals that expression in newly-formed segments is mesodermal, while expression in anterior segments is ectodermal (Additional file 5: Figure S3). It is thus clear that the stripes seen in the anterior and the posterior of the embryo, respectively, must have different function(s). The very anterior tissue between the head lobes, anterior to the mouth, expresses *sFRP125* at all stages (Fig. 5b, c, e, f). Once all segments have formed, *sFRP125* remains expressed in a segmental pattern between the limbs (Fig. 5f). Additionally, strong expression appears in a patch-like domain ventral to the base of the slime papillae and in the frontal appendages (except for their proximal region) (Fig. 5f).

*Parasteatoda sFRP125* is expressed as a faint ring at the germ disc stage (Fig. 5g). There is a dot of expression peripheral to this ring. Most likely, this expression is in the cumulus that has migrated to the edge of the germ disc (cf. [58]); the ring of expression is thus not in the periphery of the disc, i.e. the future most anterior tissue (Fig. 5g, i). When the germ band forms, *sFRP125* is expressed in the form of a broad sub-anterior domain (that appears to start splitting into two or three separate stripes), and a discrete posterior stripe anterior to the SAZ (Fig. 5j). In the gap between the anterior broad domain of splitting stripes and the posterior stripe appear to be two more very faint stripes of expression (Fig. 5j). These two stripes become stronger in the subsequent developmental stage (Fig. 5k), and the anterior domain has now split into three domains representing expression in the cheliceral (ch) segment, the pedipalpal (pp) segment and the first walking limb bearing segment (L1) (Fig. 5k). Anterior to the SAZ is now a broad domain of expression (Fig. 5k). At later stages, it becomes clear that the segmental stripes of expression are located between the now outgrowing limb buds (Fig. 5l–n). A very anterior domain has appeared, potentially associated with the soon-to-form labrum (Fig. 5l, m). Segmental expression in the last formed segments is broad and comparable to the earliest domain (ch to L1 segments) and the broad posterior domain seen at an earlier stage (cf. Fig. 5k, l/o). At late stages, expression in the labrum becomes obvious, and a complex alternating pattern of expression in the limbs appears, except for the labrum that expresses *sFRP125* only in proximal and dorsal tissue. Expression in the other appendages is restricted to dorsal tissue as well (Figs. 5p–r, 8a–f). At these late stages, *sFRP125* is also expressed in the most dorsal tissue likely representing the future heart, and as for another heart marker gene (*tinman*), there is also a V-shaped expression dorsal to the head lobes that likely contributes to the formation of the heart (Fig. 5s) (cf. [64]). There is expression in the developing brain and the mouth (Fig. 5r, s). The SAZ does not express *sFRP125* at any developmental stage (Fig. 5j–l, o, q).

**Fig. 6:**
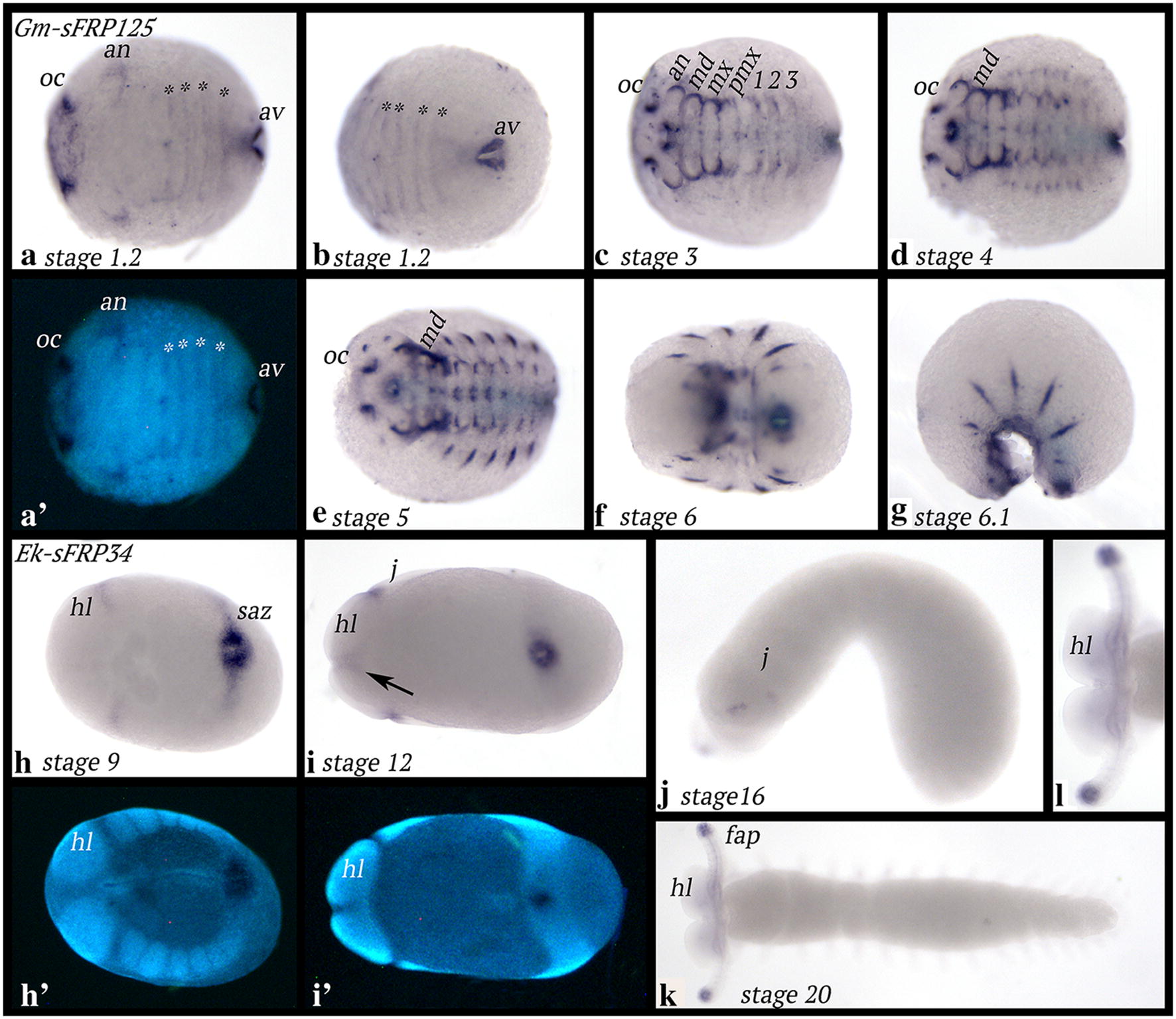
Expression of *sFRP125* in *Glomeris* (**a**–**g**) and *sFRP34* in *Euperipatoides* (**h**–**k**). In all panels, anterior is to the left. Ventral views, except for **g** and **j** (lateral views); **k**, **l** (dorsal views). **a**′, **h**′ and **i**′ Represent DAPI stained embryos as seen in **a**, **h**, **i**. Asterisks in **a**, **b** mark transverse stripes of expression. The arrow in **i** points to faint transient expression anterior to the mouth. Abbreviations as in Fig. 2

The earliest expression of *Glomeris sFRP125* is seen in the form of strong expression around the invaginating proctodaeum and weakly in the form of a broad domain of expression in the future stomodaeum. Later this anterior domain becomes stronger and two patches of enhanced expression appear on the lateral edges (Additional file 6: Figure S4). At stage 1.2, *sFRP125* transverse stripes of expression appear in the segments posterior to the position of the forming limb buds (Fig. 6a, b). At subsequent developmental stages, the initial broad anterior domain of expression disappears, except for the two dots of enhanced expression and expression in the mouth. Expression in the dorsal region of the antennae has become stronger, and the same pattern is seen in all head appendages, albeit weakly in the postmaxillary segment that does not bear any appendages (Fig. 6c). At stage 4, dorsal expression appears in the form of two domains per dorsal segmental unit; the middle of each unit does not express *sFRP125* (Fig. 6d). Expression in the form of a segmental dot per segment appears along the ventral midline (Fig. 6d). At stage 5, this expression has transformed into two parallel dots and expression in the dorsal segmental units is now in the form of a single strong stripe (Fig. 6e). This expression persists in later developmental stages (Fig. 6f, g).

### Expression of *secreted frizzled-related protein 34* (*sFRP34*)

*Euperipatoides sFRP34* is first expressed in the interface between the head lobes and the posterior adjacent jaw-bearing segment, as well as in the posterior pit region (Fig. 6h). At approximately stage 12, faint expression appears in the head lobes anterior to the mouth (Fig. 6i). At stage 16, expression in the interface between head lobes and jaw-bearing segments becomes weaker, and expression in the posterior pit has disappeared (note that at this stage, only 14 trunk segments have formed; a 15th segment will be formed later and without the presence of *sFRP34*) (Fig. 6j). Expression appears in the frontal appendages (Fig. 6j). At stage 20, *sFRP34* is exclusively expressed in the tips of the frontal appendages and what appears to be the commissures that run from there to the protocerebrum (cf. [66, 67]) (Fig. 6k, l).

We did not detect any specific signal for *Glomeris sFRP34.*

### Expression of *shifted* (*shf*)

**Fig. 7:**
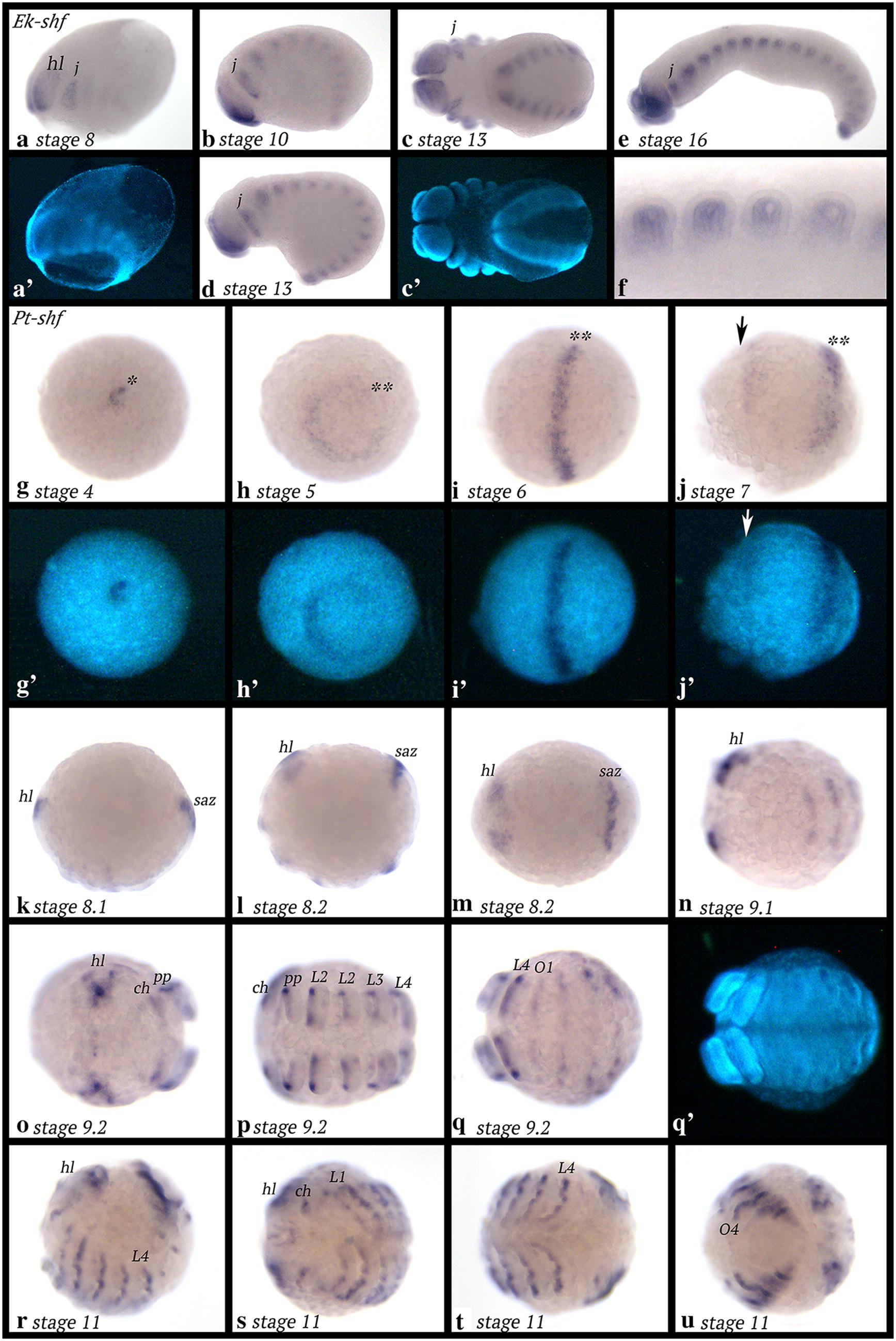
Expression of *shf* in *Euperipatoides* (**a**–**f**) and *Parasteatoda* (**g**–**u**). In all panels, anterior is to the left. Ventral views, except for panels **a**, **b**, **e**, **f**, **k**, **l** and **r** (lateral views); panel **m** (dorsal view). Panels (**a**′, **c**′, **g**′–**j**′, **q**′) represent DAPI-stained embryos as seen in panels (**a**, **c**, **g**–**j**, **q**). Asterisk in panel (**g**) marks expression in the centre of the germ disc. Double asterisks (**) follow expression in **h**–**j**. The arrow in **j** marks the anterior of the embryo. Abbreviations as in Fig. 2; O, opisthosomal segment

*Euperipatoides shf* is first expressed weakly as reflected by elongated staining time compared with other genes. Expression is in the head lobes and the jaw-bearing segment (Fig. 7a). Later, expression is in all segments in the developing appendages; this expression is mesodermal (Fig. 7b–f). There are two patches of expression lateral to the position of the jaws (Fig. 7c). Note that we observed rare cases of expression in either the posterior pit region or in broad transverse stripes of expression in the penultimate formed segment (weakly also seen in the last formed segment) (Additional file 7: Figure S5). This could reflect transient dynamic expression (or an artefact of extended staining time).

**Fig. 8:**
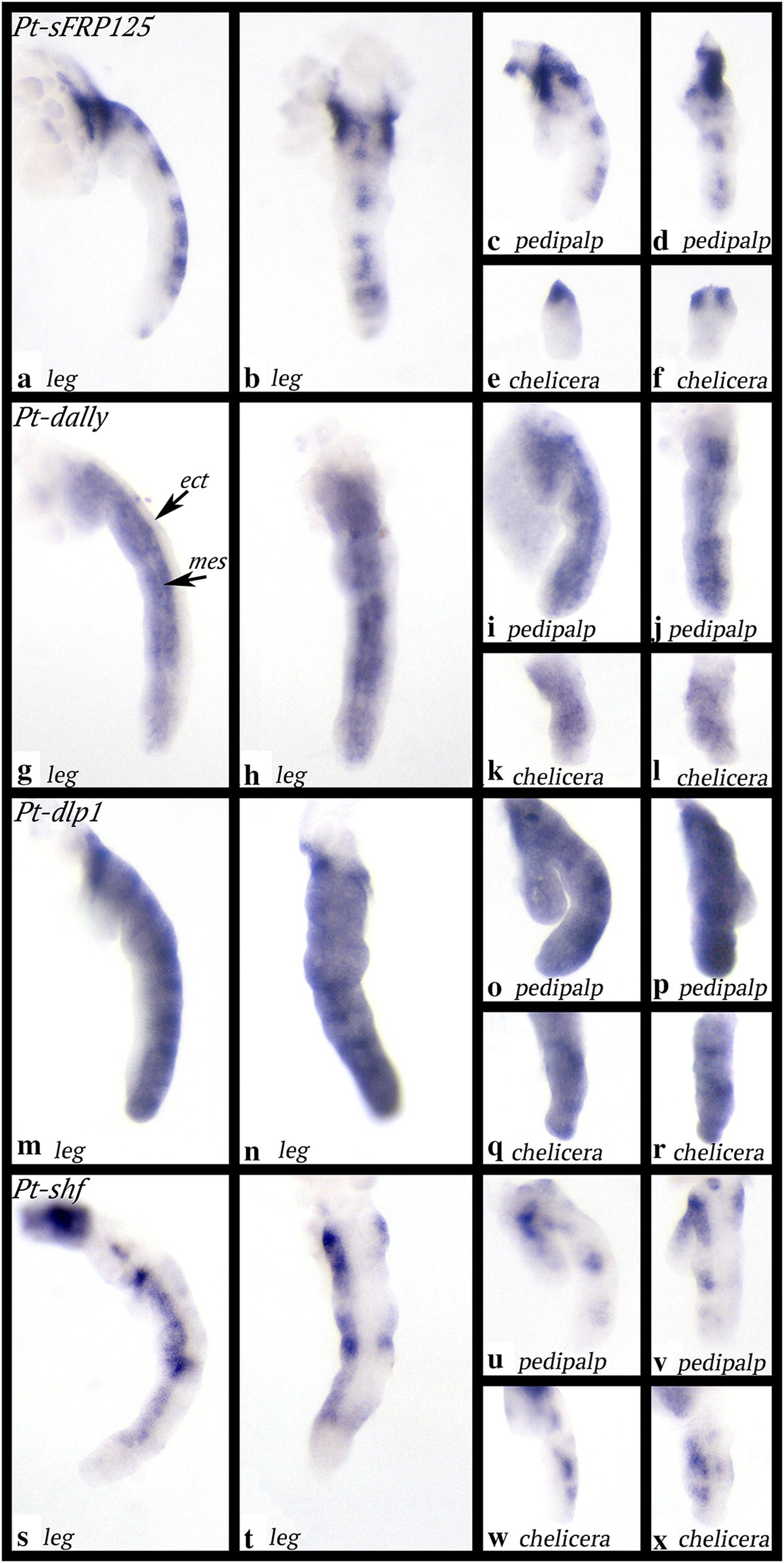
Expression of *sFRP125* (panels **a**–**f**), *dally* (panels **g**–**l**), *dlp1* (**m**–**r**) and *shf* (**s**–**x**) in dissected limbs of the spider *Parasteatoda tepidariorum*. ect, ectoderm; mes, mesoderm

*Parasteatoda shf* is first expressed in the form of a single dot in the centre of the early germ disc (Fig. 7g). In a subsequent stage, expression is in a circle close to the centre of the disc; it is currently unclear if this ring of expression develops from the earlier dot-like domain or if the latter disappears, and the ring forms de novo. As radial symmetry of the early embryo transforms into a bilaterally symmetric germ band, *shf* is expressed in a single transverse stripe in the middle of the embryo (Fig. 7i). At stage 7, a second, more anterior transverse stripe appears (Fig. 7j). At stage 8, this new stripe has become stronger; it is located in the anterior of the embryo (in the head lobes). The central stripe appears to have split into three weaker stripes corresponding to the chelicera-, pedipalp- and first walking-limb bearing segments (Fig. 7k). At the same time, expression appears in the form of a transverse stripe in the SAZ (Fig. 7l, m). At a later stage, there are transverse stripes of *shf* expression in every segment (Fig. 7n–q). Expression in the head lobes becomes complex (Fig. 7o). Expression in the appendages is along the ventral side. This expression is stronger in the anterior sector (Figs. 7o–t, 8s–x), and weaker in the posterior sector; the middle of the ventral region of the appendages does not express *shf* (Fig. 8). In the opisthosoma, *shf* is expressed in dorsal tissue (Fig. 7r, u).

**Fig. 9:**
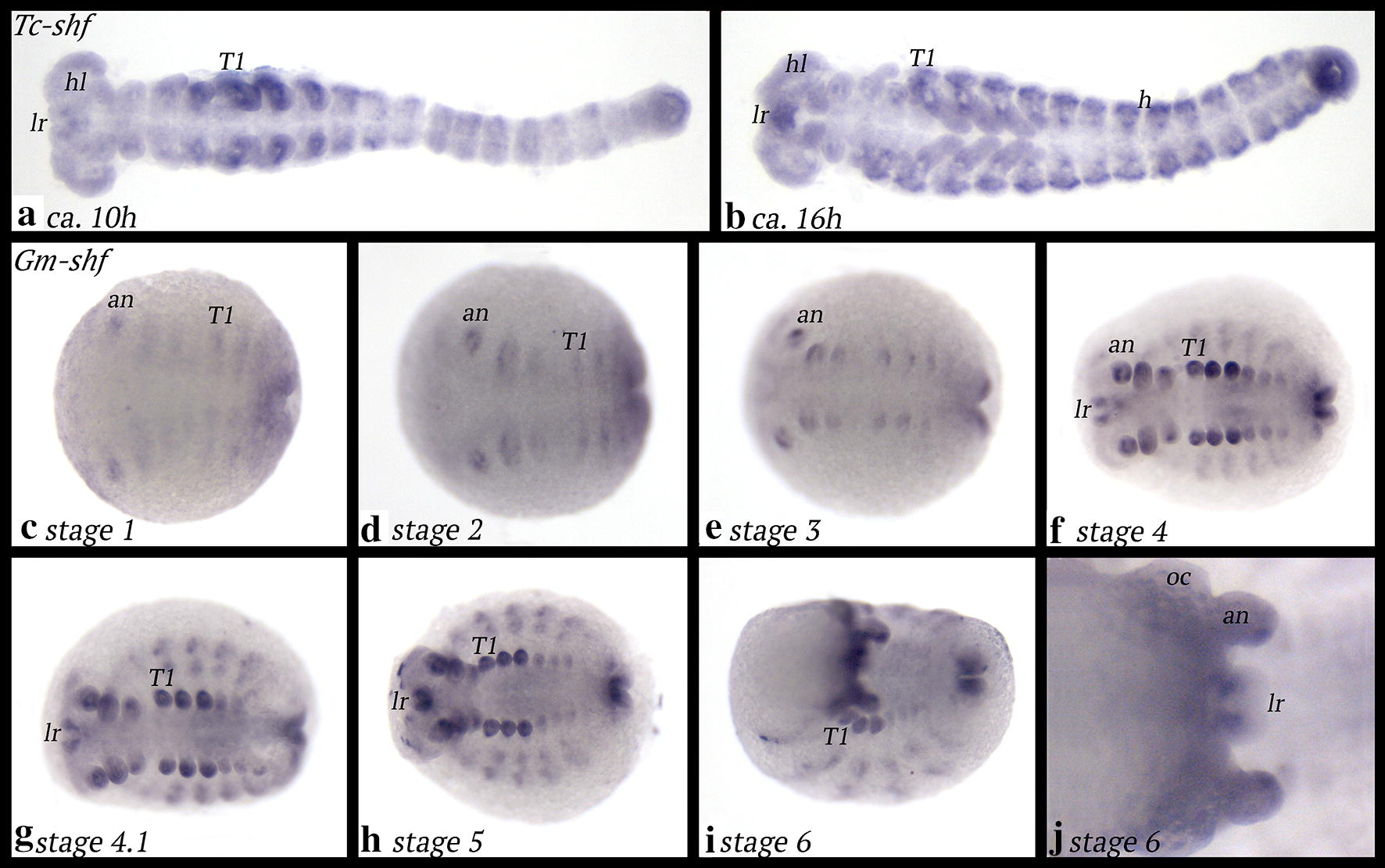
Expression of *shf* in *Tribolium* (**a**, **b**) and *Glomeris* (**c**–**j**). In all panels, anterior is to the left. Ventral views, except for panel **j** (dorsal view). Abbreviations as in Fig. 2

*Tribolium shf* is expressed in the mesoderm of the appendages, the abdominal segments (Fig. 9a, b) and dorsal mesoderm that may contribute to the development of the heart (Fig. 9b) (cf. [64]).

*Glomeris shf* is expressed as transient segmental stripes in early developmental stages (Fig. 9c). Later, expression is restricted to the appendages and the anal valves (Fig. 9d–i). In dorsal tissue, *shf* is expressed as a broad stripe with enhanced expression proximally and distally (Fig. 9f–i). Expression in the labrum is proximal, but in the antennae, expression is in the form of a dot ventrally in the tip (Fig. 9j).

## Discussion

### Comparison of embryonic gene expression patterns reveals little conservation suggesting the possibility of MMFs representing key regulators in evolution

Arthropods have evolved in numerous different shapes and forms, and each species possesses unique body features, each of which is the result of different interaction of their genetic toolkit(s). The interaction and fine-tuning of gene function is likely a key factor in evolution. Morphogens clearly represent important factors in development and evolution, and still there are only relatively few morphogen signalling pathways, and their components are often expressed in rather conservative patterns. The question is how these few and conservatively expressed genetic factors can be regulated to possibly contribute to the plethora of different forms and thus functions in development (and evolution).

**Fig. 10:**
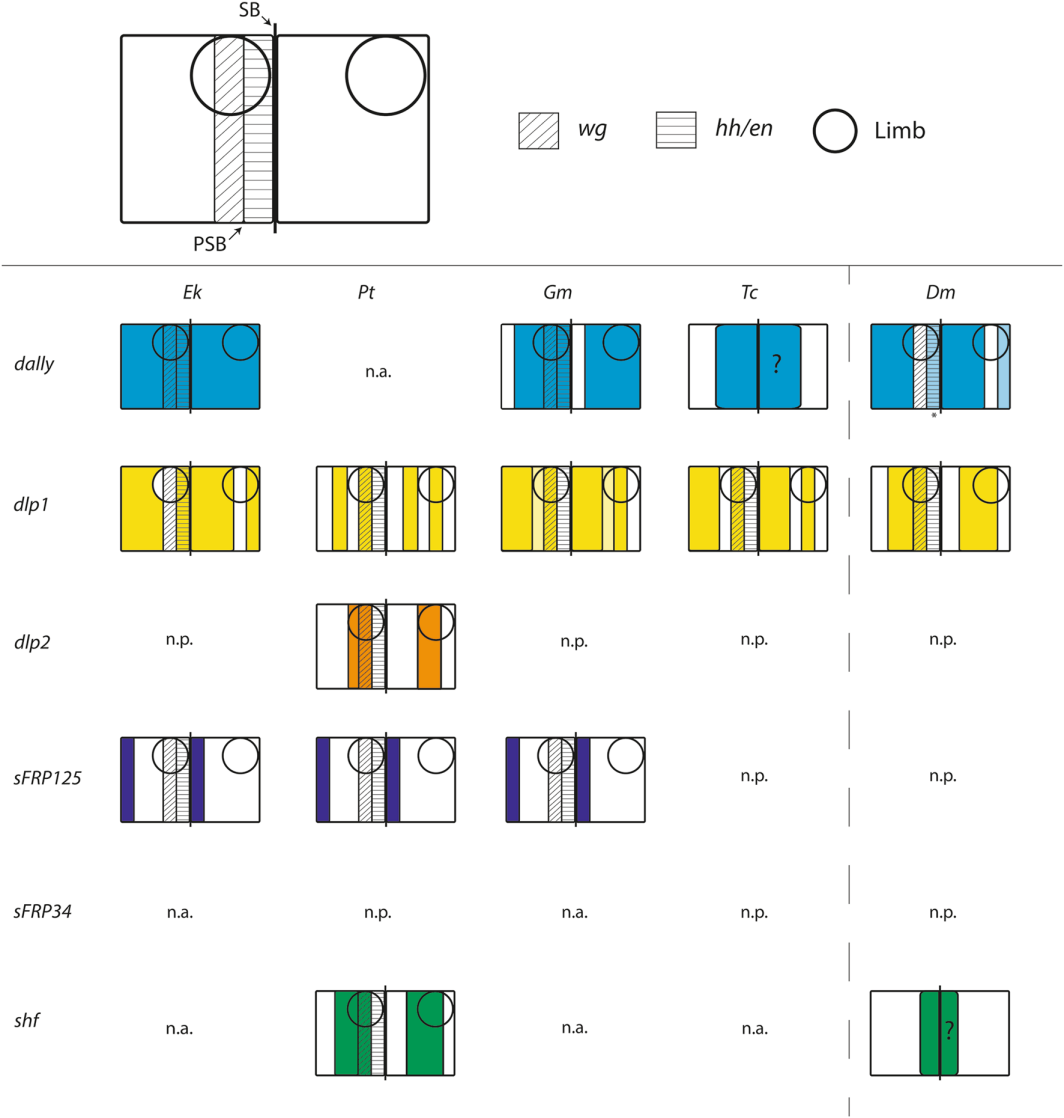
Schematic summary of striped segmentally reiterated patterns of expression in panarthropods. Abbreviations: Dm, *Drosophila melanogaster*; Ek, *Euperipatoides kanangrensis*; Gm, *Glomeris marginata*; Pt, *Parasteatoda tepidariorum*; Tc, *Tribolium castaneum*. Abbreviations: n.a., not applicable (expression is not in segmental stripes); n.p. not present (ortholog is missing from the genome/transcriptome). Questions marks indicate unclear intra-segmental position of expression (for *Tc*-*dally* and *Dm*-*shf*)

The embryonic expression profiles of most of the MMFs investigated here, such as *dally* differ among all investigated species, and there is only little potentially “conserved” patterning. *dlp* is expressed in transverse stripes, especially during stages of segment patterning and addition, and in tissue that will likely develop into the heart, while it is never expressed in the SAZ. The level of transcriptional conservation is thus much higher than that of *dally*. However, the patterns of the two *dlp* paralogs in the spider are partially complementary suggesting a novel function of *dlp2* in a region where *dlp* is not upregulated in the spider (*dlp1*) and the other species (Fig. 10). In all investigated species, *sFRP125* is expressed in transverse segmental stripes indicating a specific and conserved function in segmentation. Additionally, there is expression in the head in all species, likely associated with a function in brain development. However, many of the expression patterns are unique for each given species such as the segmental expression of *sFRP125* in the appendages of the spider or the fact that segmental stripes of expression in newly-formed segments in the onychophoran are mesodermal, not ectodermal as it is the case for the arthropod species (and for similar stripes in later stage onychophoran segments). *sFRP34* has been lost in insects [68] and the spider, and in the millipede, there is no detectable embryonic expression, while in the onychophoran *sFRP34* is strongly expressed in specific regions of the developing embryo. The expression profiles of *shf* are very diverse and there are no obvious similarities that could be interpreted as evolutionary conserved patterns suggesting that the role of *shf* is different in each of the investigated panarthropod species.

Together these data suggest that MMFs may indeed contribute significantly to modifying morphogen signalling pathways that are otherwise embedded in highly conserved genetic networks (the interaction of the morphogen(s) with its receptor(s) and the activation/repression of conserved downstream factors). MMFs may thus represent components of the genetic toolbox that appear to be free to evolve and thus allow for different regulation of morphogen signalling rather than the morphogens themselves and their receptors, all which are expressed in rather conserved patterns (e.g. [21, 22, 29, 39]).

### Potential interaction and function of MMFs in segmentation

In *Drosophila*, other arthropods, and likely even in onychophorans, Wg- and Hh-signalling interact in a highly conserved autoregulatory loop to specify and maintain segment and parasegment boundaries (e.g. [21–23, 26, 28, 32, 34, 69–71] (note that these authors interpret their data differently [71])). Cells posterior in the segment express the transcription factor *engrailed* (*en*) that activates expression of *hedgehog* (*hh*). Hh protein is secreted from these cells, and signals to adjacent anterior cells by binding to its receptor Patched (Ptc). Binding of Hh to Ptc leads to the transcription of *wg* expression through Cubitus-interruptus (Ci). Wg protein is secreted from these cells and signals to the posterior adjacent *en*-expressing cells which express Fz receptors to which Wg binds. Binding of Wg to Fz (re)activates the expression of *en*. Many of the genes involved in this autoregulatory loop are the so-called segment-polarity genes (SPGs) because of their mutant phenotypes. What most of these genes have in common is that they are expressed in distinct and highly conserved patterns, typically in transverse segmentally reiterate stripes (e.g. [72]).

*Drosophila dally* is expressed in *en*-expressing cells as indicated by enhancer trap lineages, but its strongest expression is in cells anterior to *wg*. *dally* is thus expressed in non-*wg*-expressing cells [43, 73]. Expression in the spider does not indicate a role in segmentation as it is the case for *Drosophila*, and the ubiquitous expression in *Euperipatoides* is not informative in this context because the level of posttranscriptional regulation of *dally* is unclear. Expression in *Glomeris* and *Tribolium* is in segmental stripes similar to that of *Drosophila*, although our double-staining data (Additional file 3: Fig. S1) indicate that *dally* is expressed in the complete segment except for cells posterior to *en* (Fig. 10). There is thus flexibility in *dally* expression (at least at the mRNA level).

*Drosophila dlp is* expressed anterior to *en*, overlapping the domain of *wg* expression, and in a few cells anterior to that [74, 75]. In contrast, onychophoran *dlp* is expressed in *en*-expressing cells and cells posterior to *en*, and thus rather in a pattern like *Drosophila dally* (Fig. 3). In *Tribolium*, *Parasteatoda* (for *dlp1*) and potentially also in *Glomeris*, *dlp* is expressed in two stripes per segment although expression appears first as one broad domain in nascent segments in *Glomeris*. This broad stripe then appears to transform into two by central fading of expression; this central area is likely where *wg* is expressed.

*sFRP125* genes are expressed posterior to the distinct transverse segmental stripes of *en*-expressing cells in all investigated arthropod species (note that there are no sFRPs in insects). Since sFRPs interfere negatively with Wnt-signalling in vertebrates [76–79], it is possible that this function is conserved in panarthropods and used to prevent Wg-signalling in cells posterior to *en*-expressing cells. In the onychophoran, the ectodermal (anterior stripes) expression of *sFRP125* is overlapping with the posterior region of *en*-expression which appears to be specific for onychophorans but not the anterior which is like in arthropods in the posterior of the limb buds (cf. [32, 71]). The function of *sFRP125* could thus still be to prevent Wg-signalling reaching too far posteriorly.

Although the vertebrate *WIF1* gene (*shifted* (*shf*) in *Drosophila*) negatively regulates Wnt-signalling [80], this function is not conserved in *Drosophila*. Instead, *shf* positively interacts with Hh-signalling [15, 16]. Expression pattern analysis in the embryonic ectoderm during segmentation is scarce but *shf* is shown to be expressed in the form of transverse segmental stripes [15].

It is only in the spider *Parasteatoda* that expression of *shf* suggests a possible function in segmentation. Interestingly, *shf* is first expressed in the form of a single dot at the early germ disc stage. This expression is similar to that of *hh* and its receptor *ptc* in the blastopore, that later contributes to the SAZ [37] indicating involvement with Hh-signalling. However, there is also expression of a Wnt gene, *Wnt11.2*, in the early forming SAZ (Janssen et al. [29] (in the supplementary data)) indicating possible interaction with Wnt-signalling. This is further supported by the expression of *Wnt11.2* in the prosomal appendages in *Parasteatoda*, very much resembling the late expression of *shf* [29]. The successive appearance of expression of *shf* in the form of transverse segmental stripes in the early germ disc and early germ band resembles that of *hh* rather than *wg* (which is expressed later; note that several Wnt genes are expressed in at least one broad anterior domain in the germ disc) [29, 31].

Altogether, expression of some of the potential MMFs investigated here in segment-polarity gene like reiterated transverse stripes indicates involvement in segmentation. However, the sparse (or indeed lacking) published data on these genes in any arthropod except for *Drosophila*, together with their interaction with multiple morphogens such as Hh and Wnts, impedes interpretation of our data. Further research is needed to identify the exact position of MMF expression within the segments, and functional analyses then have to be conducted to reveal the exact interaction of the MMFs with one or more given morphogens.

### Different patterns of MMFs in dorsal versus ventral segmentation in *Glomeris*

**Fig. 11:**
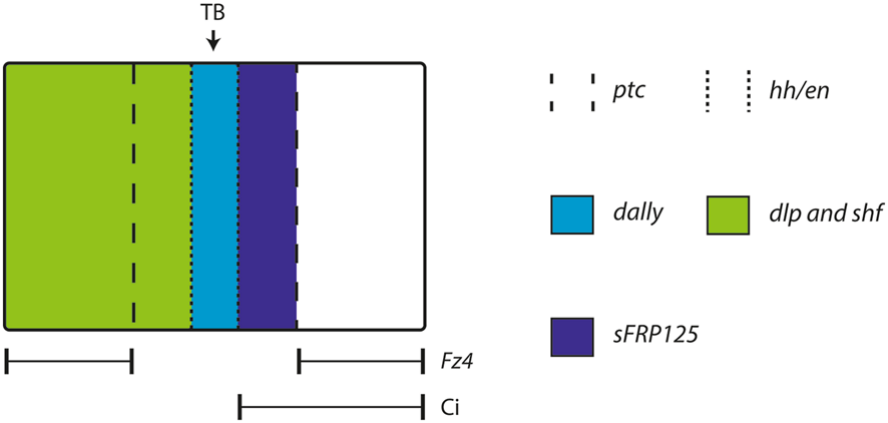
Schematic overview over gene expression of MMFs in the dorsal segmental units of the millipede *Glomeris marginata*. *Dally* is co-expressed with *hh*, *en* and *ptc*. *ptc* is expressed on either side of *en*/*hh*/*dally*. Posteriorly, *ptc* is co-expressed with *sFRP125* that may interact with the binding of Hh to Ptc. Anteriorly, *ptc* is co-expressed with *dlp* and *shf*, possibly interacting with Hh. Either of the three MMFs, *dlp*, *shf* and *sFRP125* could serve to break the possible symmetry of Hh-signalling. TB, tergite border

*Glomeris* offers the opportunity to study AP body segmentation in ventral and dorsal segmental units. Earlier research has shown that the interaction of SPGs is likely conserved in ventral segmentation. A similar set of genes (SPGs) also acts in dorsal segmental units (e.g. *en*, *hh* and *ptc*), but there the genes do not appear to be involved in segment border formation, but rather the establishment of the borders between the dorsal armoured plates (tergites) covering the back of millipedes [23, 28, 81, 82]. In dorsal tissue, Wnt-signalling does not seem to be involved; at least *wg*, the Wnt gene that is a conserved key factor in ventral segmentation in arthropods, is not expressed in dorsal tissue in *Glomeris* [28]. Other Wnts, however, are expressed in dorsal tissue, and it may be that they are involved in the formation of tergite boundaries [29, 35]. Interestingly, all investigated MMFs, except for *sFRP34*, are expressed in specific patterns in dorsal segmental units suggesting interaction with morphogen signalling; this may either be Hh-, Wnt- or Dpp-signalling. The most prominent dorsal OC is the *en* and *hh*-expressing region in the middle of the dorsal segmental units where the tergite boundaries form. Interestingly, *dally* is co-expressed with *en*/*hh* (Additional file 3: Figure S1) and *dlp* and *shf* appear to be expressed anterior to this region, while *sFRP125* appears to be expressed posterior to *en*/*hh*/*dally*-expressing cells. Whatever the function of MMFs may be in tergite border formation, it seems likely that they contribute to defining sharp borders between *en*/*hh*-expressing cells, and anterior as well as posterior adjacent cells. Since the Hh receptor *ptc* is expressed on either side of *en*/*hh*, signalling could be bidirectional (although *ci*, a mediator of Hh-signalling is restricted to anterior tissue [28]). *sFRP125*, *shf* and *dlp* could therefore also be involved in breaking the possible symmetry of Hh signalling (Fig. 11).

### Posterior elongation

Posterior elongation of the AP axis and segment addition are two morphologically closely-linked processes (e.g. [31, 34, 36, 70, 83–85]). Here Wnt-signalling, Caudal and Delta/Notch-signalling interact in a gene regulatory network that controls posterior elongation (reviewed in [55, 86]). Most of the data showing that Wnt-signalling is involved in this process come from data on *Wnt8* and *wg*/*Wnt1* (reviewed in [55]), but of course that does not exclude the possibility that other Wnt ligands may be involved as well. And indeed, in many arthropods, levels of redundancy of Wnt ligands appear to exist (e.g. [21, 49, 70, 87]). In the species investigated here, multiple Wnt ligands are expressed in the SAZ or posterior to that in the posterior pit/anal valve region [21, 24, 29, 35, 70]. Similarly, many of the MMFs display specific expression patterns in the SAZ and posterior to that, as for example the absence of expression of *dlp* in the SAZ of *Euperipatoides*, *Glomeris* and *Tribolium*; or the absence of expression of *sFRP125* from the posterior part of the SAZ in the spider, or the distinct expression of *sFRP125* in the anal valves in *Glomeris*, or of *sFRP34* in the posterior pit in *Euperipatoides*. These data imply that the MMFs have specific functions in morphogen regulation, possibly via the interference with Wnt-signalling, one of the key factors of posterior elongation and the addition of segments.

### Potential interaction and function of MMFs in arthropod and onychophoran limb development

Evolution of the jointed limbs represents one of the key topics of arthropod evolutionary developmental research. This is because the limbs of arthropods likely represent one of the key innovations of this group of animals responsible for their great evolutionary success leading to immense morphological variation. This becomes especially obvious in comparison to the very uniform morphology of the few hundreds of extant species of onychophorans, which do not possess jointed limbs.

In arthropods, the limbs are patterned along three morphological axes, the anterior to posterior axis (AP), the dorsal to ventral axis (DV) and the proximal to distal axis (PD). The expression of genes responsible for coordinated limb axes formation are well preserved among different classes of arthropods, although most (especially functional) data still come from the model system *Drosophila melanogaster* (reviewed in [88]). Wnt- and Hh-signalling play pivotal roles in the development of the limbs. The AP axis is under control of the morphogens Wingless (Wg/Wnt1) and Hedgehog (Hh) (e.g. [89, 90]), while the PD axis and the DV axis are determined by *inter alia* the function of Wg and another morphogen, Decapentaplegic (Dpp) [89, 91–93].

In *Drosophila*, *wg* is expressed in the central and ventral region of the developing limbs, and in other arthropods this expression is conserved (e.g. [28, 30, 69]), suggesting conserved function. These data imply that there is a need for restriction of the source of Wg production (the *wg*-expressing cells) to the central and ventral region for DV and AP axis formation interacting with Dpp and Hh, respectively.

In AP axis formation, Wg interacts with Hh in posteriorly adjacent cells [cf. the role of these genes in the maintenance of segmental (parasegmental) boundaries in body segmentation (discussed above)].

In PD axis formation, Dpp and Wg form distal to proximal activity gradients that regulate the expression of target genes in concentric rings along this axis [94], and in DV axis formation Dpp and Wg function as dorsal and ventral morphogens, respectively [95, 96]. While *dpp* and *wg* are expressed along the complete dorsal and ventral ectoderm of the legs, respectively, in *Drosophila*, the topology of the direct developing legs of most other arthropods likely requires a modification of the expression pattern(s) of *dpp* and *wg*. The so-called topology model offers a logical explanation for potentially conserved function of Wg and Dpp in the two-dimensional limb disc of *Drosophila* and the three-dimensional directly developing limbs of arthropods (discussed in [97]). The model requires that the source of one of the two morphogens, Dpp or Wg, must be restricted to the tip region, and from there form a gradient along the PD axis of the limb.

In the onychophoran *Euperipatoides kanangrensis*, both *dpp* and *wg* are expressed in the tips of the legs and thus the requirements for a topology model-based interaction of Wg and Dpp are present ([32, 42, 71]; see [98] for a different pattern of *dpp* expression in the onychophoran *Euperipatoides rowelli*). It is likely that a PD morphogen gradient exists in the onychophoran legs because the putative target genes of such a gradient, the so-called leg gap-gene orthologs, are indeed expressed in concentric rings regionalizing the PD axis [99], very much as it is the case in *Drosophila* and other arthropods. Restriction of the source of both Dpp and Wg to the tips of the appendages would not create any struggle for the formation of a PD gradient as long as they are both transported from their source of transcription (and translation) along the PD axis of the leg. The absence of a ventrally-restricted domain of Wg, however, would require different regulatory mechanism in DV and AP leg axis patterning.

**Fig. 12:**
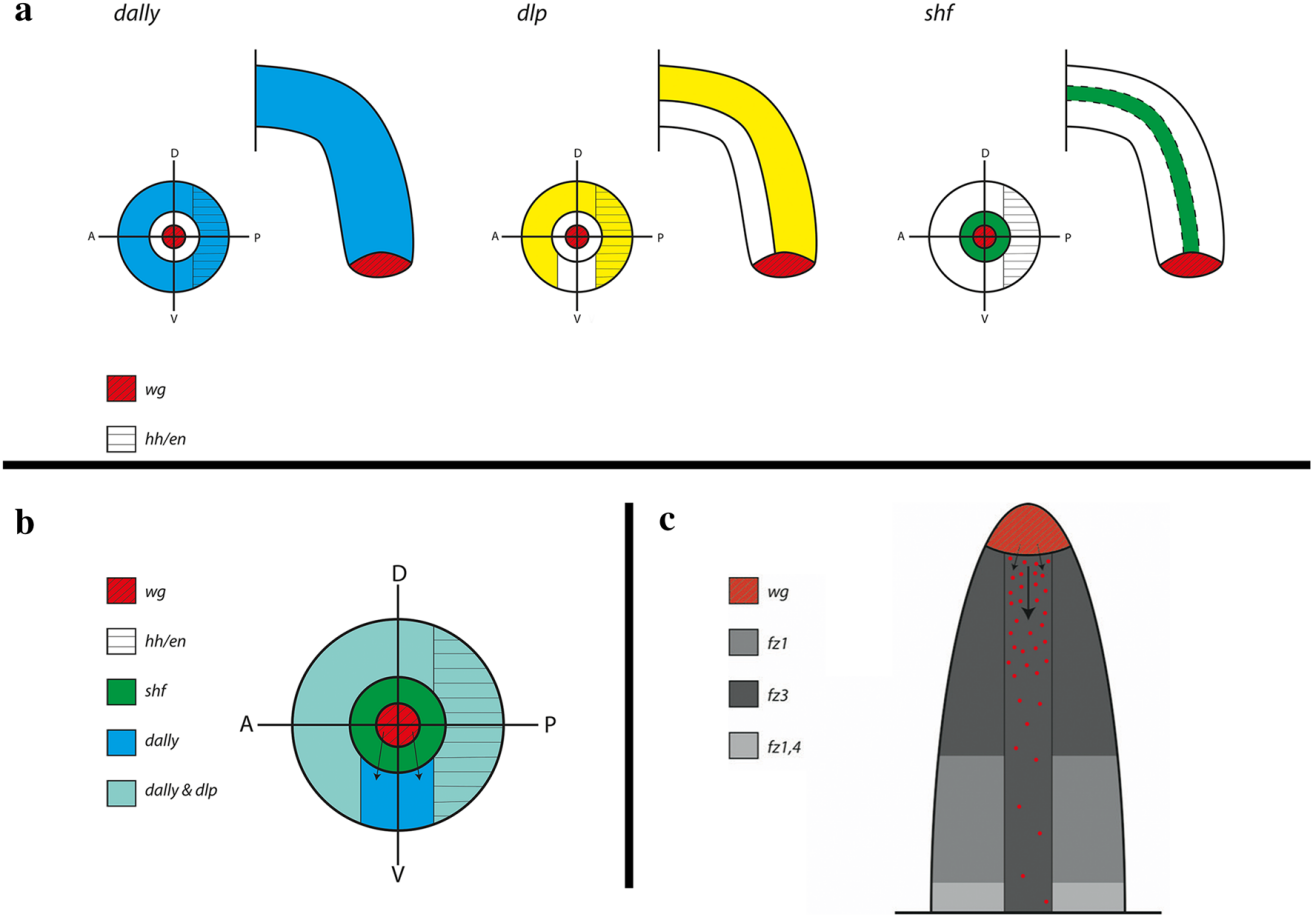
Schematic overview over gene expression of MMFs in the legs of the onychophoran *Euperipatoides kanangrensis*. **a** Expression patterns of *dally*, *dlp*, and *shf* in correlation to expression of *wg* in the tips of the limbs, and *engrailed*/*hedgehog* (*en*/*hh*) expressing tissue. Depicted are the legs in ventral view (top) and in cross section (bottom). In the latter, the outer ring represents ectoderm, the middle ring represents mesoderm and the inner ring represents the *wg*-expressing tip of the leg. Dorsal (D), ventral (V), anterior (A) and posterior (P) are indicated. The dashed line in the ventral view on a leg expressing *shf* indicates the mesoderm. **b** Summary of gene expression of *dally*, *dlp* and *shf* in a given leg, cross section. The arrows mark suggested direction of Wg morphogen travelling in the dally-corridor. **c** Expression of Frizzled receptors in comparison to *wg* expression. The arrows mark suggested direction of Wg morphogen travelling in the dally-corridor; note that *fz3* is expressed in this corridor as well, while other Fz genes are not

The differential expression pattern(s) of some of the MMFs investigated here offer explanations of how a ventrally-restricted (or ventrally enhanced) distal to proximal gradient of Wg could be established in the onychophoran leg (Fig. 12): The source of Wg is in the tip of the leg. From there, Wg could theoretically diffuse through the extracellular space to form a uniform PD gradient in dorsal, ventral, anterior and posterior ectodermal tissue. More likely, however, is that Wg requires an active transport through the extracellular space, likely by means of interaction with the membrane bound glypicans Dally and Dlp. Since *dally* is expressed ubiquitously in all ectodermal cells that do not express *wg*, transport via Dally alone would not contribute to a ventral gradient. Expression of *dlp* is restricted to all ectoderm except for the ventral corridor of the developing limb (Fig. 12); it is therefore impossible that Dlp transports Wg along the ventral side of the limb. However, binding of Wg to glypicans is not necessarily a Wg-signalling promoting process (reviewed in, e.g. [7]). In contrast, Wg could be bound by either of these glypicans but not transported. If Dlp does so, then transport of Wg via Dally would form a ventrally-restricted long-range PD gradient of Wg (especially if the affinity of Dlp to Wg is higher than that of Dally to Wg, as it is the case in the *Drosophila* wing disc (e.g. [7]), resulting in a scenario comparable to that in *Drosophila* and other arthropods (Fig. 12). Also, in the *Drosophila* wing disc, Dlp is suggested to function as a competitor of morphogen binding with their receptors [100]; it is thus not unlikely that Dlp competes with Fz(s) in those areas where *dlp* is expressed, further supporting the idea that Wg is exclusively transported along the ventral sector of the leg PD axis where *dlp* is not expressed. Ventral Wg could then interact with Hh expressing cells in AP axis formation and maintenance as it does in arthropods, and it could fulfil a function as a ventral morphogen, again as it is the case in *Drosophila* and (likely) arthropods in general.

The (weak) expression of the hydrolase Notum along the ventral side of the leg [26] does indeed indicate that Wg-Dally complexes exist in this ventral tissue; a function of Notum in *Drosophila* is to fine-tune Wg-signalling by cutting the connection of Wg and Dally and thereby negatively regulating Wg-signalling [101, 102]. If this function is conserved in the onychophoran, then expression of *Notum* only makes sense if Wg-Dally is present there as well, as it would according to the scenario suggested here.

The other potential MMFs investigated here, *shf*, *sfrp125* and *sfrp34*, are not expressed in the ectoderm of the developing onychophoran legs providing a relatively simple interaction of MMFs with Wg-signalling.

**Fig. 13:**
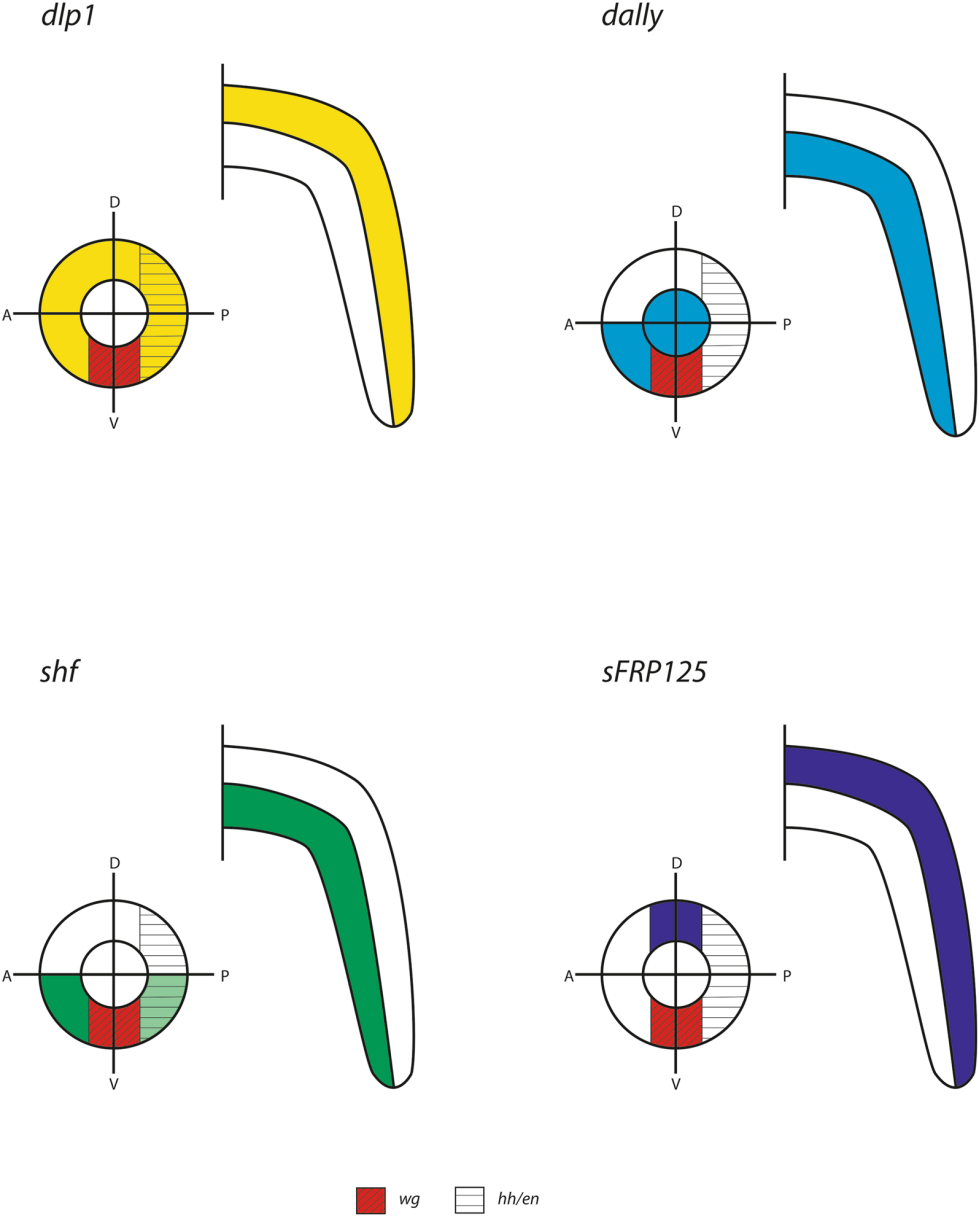
Schematic overview over gene expression of MMFs in the legs of the spider *Parasteatoda tepidariorum*. See legend Fig. 12 for further details

In the arthropod legs, exemplified by gene expression patterns in the spider *Parasteatoda*, the interaction of MMFs appears to be more “complex”, potentially reflecting the more “complex” morphology of these appendages compared to the relatively “simple” tube-like legs of onychophorans (Fig. 13). In *Parasteatoda*, as in other arthropods, *wg* is expressed along the ventral side of the leg [29], and *dpp* is expressed in the tip of the leg [41] (and its downstream target *optomotor*-*blind* (*omb*) is expressed in all dorsal ectodermal tissue [103]) suggesting conserved interactions in DV and PD axis development. Also, *wg* is expressed anterior adjacent to *engrailed* (*en*) and *hh* providing conserved interactions in AP axis development. However, the domain of *wg* expression is surrounded by potential regulators and modifiers of Wg-signalling. Assuming that Wg needs glypicans for long-distance transport, such transport is possible because *Parasteatoda dlp1* is expressed in all ectodermal tissue except for the *wg*-positive cells (Fig. 13) (as in the onychophoran). Additionally, *dally* is expressed posterior adjacent to *wg* and in the mesoderm of the leg (Fig. 13). It is likely that MMFs are needed to restrict and/or fine-regulate the activity of Wg. For example, *shf*, a negative regulator of Wg-signalling in *Drosophila* is strongly co-expressed with *dally* in the anterior ventral region of the legs, possibly preventing or down-regulating Wg-signalling into this tissue. Conversely, *shf* is only weakly expressed in tissue posterior to *wg*, the tissue that also expresses *en* and *hh* and thus the target of Wg-signalling. Spreading of Wg into the mesoderm that expresses *dally* as well appears to be restricted by a strong domain of *Notum* expression in the ventral mesoderm of the leg (note that these data come from another spider, *Cupiennius salei* [25]). The secreted Frizzled-Related Protein encoding gene, *sFRP125*, is expressed in the dorsal central sector opposite of the *wg*-expressing sector. *sFRP125*, however, is not expressed in a continuous PD dorsal domain, but in patch-like dorsal domains, potentially restricting Wg-signalling to act in distinct regions of the dorsal ectoderm.

## Conclusions

Regulation of morphogen function is complex and relies on the interaction of multiple factors, many of which, like the MMFs investigated here, have multiple functions, can interact with multiple different morphogens, and can have opposing effects based on genetic context and morphogen concentration. Differences in morphogen function have been reported based on genetic context between species, but also in a given species. This high degree of regulatory flexibility of morphogen function is reflected by the expression patterns of the MMFs. The level of conservation is relatively low as suggested by divergent expression patterns in the different species. Therefore, this study cannot serve as anything other than a first step into investigating MMFs in these emerging panarthropod model species. Subsequent studies are needed to investigate gene function by means of knock-down experiments. Such experiments are currently not possible for any onychophoran or myriapod species, but can be conducted in the spider *Parasteatoda* and the beetle *Tribolium* (e.g. [104, 105]).

Despite the above caveats, our data clearly indicate involvement of MMFs in morphogen signalling, and that these factors partly play roles in limb development and body segmentation, two of the main research field of (pan)arthropod evolutionary developmental research (EvoDevo).

## Additional files

**Additional file 1: Table S1.** Primer list.

**Additional file 2: Table S2.** Accession numbers.

**Additional file 3: Fig. S1.** Expression of *Glomeris dally* + *engrailed* (*en*). Abbreviations as in Fig. 2. Ventral views. The asterisk in **c** marks a single distinct stripe of enhanced expression in a dorsal segmental unit; this shows that *en* and *dally* are co-expressed (with no or minimal overlap of one gene’s expression compared to that of the other). Note that expression of *en* overlaps with the posterior of the *dally*-expressing domain in ventral segmental units.

**Additional file 4: Fig. S2.** Expression of *Tribolium dlp* in the legs and the antennae. Asterisks mark rings of expression in the legs. The arrowhead marks expression in the tip off the antenna. Abbreviations: an, antenna; T1, first thoracic leg.

**Additional file 5: Fig. S3.** Confocal microscopy of *Ek*-*sFRP125*. Stage 13 embryo. Anterior to the left, dorsal up. The embryo is stained with FastRed (for *sFRP125*) and DAPI (for DNA). Optical sections were taken every 6.5 μm. A Z-stack. The arrow points to signal in L13 (mesodermal). The arrowhead points to signal anterior in L6 (ectodermal). In all panels, arrow and arrowhead point to identical position. A′ Optical section z-08. Strong signal in L13, no signal in L6. A´´ Optical section z-14. Disappearing signal in L13 (quenched by overlying DAPI signal (ectodermal cells)). Appearing signal in L6 (no overlying DAPI signal). A′′′ Optical section z-25. Signal in L13 disappeared. Strong DAPI signal (ectodermal cells). Strong signal in L6. Abbreviations: fap, frontal appendage; j, jaw; L, leg; sp, slime papilla.

**Additional file 6: Fig. S4.** Additional aspects of *Glomeris sFRP125* expression. Abbreviations: p, proctodaeum; (s), primordium of the stomodaeum. See main body text for further information.

**Additional file 7: Fig. S5.** Additional aspects of *Euperipatoides shf* expression. Abbreviations as in Fig. 2. Asterisks mark a stripe-like domain in the penultimate newly-formed segment (**a**, **b**, **d**) and in the posterior pit region (**c**). This expression was not seen in all embryos stained for *shf* and it may represent a dynamic and transient domain of expression, possibly involved in segment formation and/or patterning.
