# Supplementary material for: Gene expression analysis of potential morphogen signalling modifying factors in Panarthropoda
Source: EvoDevo. 2018 Sep 29;9:20. doi: 10.1186/s13227-018-0109-y (PMC6162966; doi:10.1186/s13227-018-0109-y)
Supplement: Supplementary file 1 — Additional file 1: Table S1. Primer list. [file 13227_2018_109_MOESM1_ESM.docx]

| Gene Name | Forward Primer | Backward Primer |
| --- | --- | --- |
| *Ek-dally* | ATGGTTCCAGAAACGCCAGTAA | AAGTTAGCTGTTGAAGGAGGAG |
| *Pt-dally* | TGTTACCGCATCTTCTCTCAAC | GAACTATTTGTTGGAGACGCCT |
| *Gm-dally* | TTCGGTGTTGTGATGGTGTTCT | ACAGCCAATGTTTCATCGTTGC |
| *Tc-dally* | TTAAGGAAGCAAGGACAAAAGG | GAAGCTCTCATCCTTGCATAGA |
|  |  |  |
| *Ek-dlp* | TGGGAAGGCAATACAAGGTGAT | GGCTCATCATCTTTACCTCCAA |
| *Pt-dlp1* | GTCCCTATCCAAGCAATATCTG | GAGGATTATTGCACATGAAGCC |
| *Pt-dlp2* | TGAAAGAGGACTGACTTGTTGC | GTCTAGCTCCAAGGTTATGCAA |
| *Gm-dlp* | CTCAAAGGATTCAGCAGCAAGT | TAGATGAGACCAAAACGACCGA |
| *Tc-dlp* | AACTGGAGACGTACTATAACCG | CTTTGGACAGCGACATCTTTGC |
|  |  |  |
| *Ek-sFRP125* | AGAATCGGCTATGGAAAGATGC | ACCACGCCCTTCTTTACCTGAT |
| *Pt-sFRP125* | ACAGTTACATAACGGAATGGGG | GTTCCACTTTTATGATGTCCTC |
| *Gm-sFRP125* | CCGCAGCACAACAGCCATTTAT | TTTTGCGGGTCCTTCGATTGGT |
|  |  |  |
| *Ek-sFRP34* | ATTGAAACTATGGAAAGAGCGG | TTCTTCTTTCCTTTGATGGCGT |
| *Gm-sFRP34* | TTATGATGATTTGGACTCGCTG | TTTCATCAACGGCTCGCATCCT |
|  |  |  |
| *Ek-shf* | TTTGTCATCACAGAAGGTCGAG | ATGCACCACCATAACGTCTGTT |
| *Pt-shf* | TGATGGAATGTTTTGTCGGTGC | ACGCACTTGTGGTTTTCGCAAA |
| *Gm-shf* | ACCAGTCGCATTTACTTGATGG | CTTCGTGTTGGGAGAACCTTTC |
| *Tc-shf* | TAAAAACGACCCCCGAAATAAC | TGCTTCTGTGCGGTTTTGCCTT |
|  |  |  |
| *Ek-smo* | CGGCTTTGGAGAGGTTTACAGA | GGAAGAAGAACAAACACAACCC |
| *Gm-smo* | GATGGACTCTGCCCTACTCTTT | ACGCTAAAGTGGTGGCTACGAT |
