# Supplementary material for: Gene expression analysis of potential morphogen signalling modifying factors in Panarthropoda
Source: EvoDevo. 2018 Sep 29;9:20. doi: 10.1186/s13227-018-0109-y (PMC6162966; doi:10.1186/s13227-018-0109-y)
Supplement: Supplementary file 2 — Additional file 2: Table S2. Accession numbers. [file 13227_2018_109_MOESM2_ESM.docx]

| Gene Name | Accession Number |
| --- | --- |
| Ek-dally | LS991957 |
| Pt-dally | LS991958 |
| Gm-dally | LS991959 |
| Tc-dally | LS991960 |
|  |  |
| Ek-dlp | LS991961 |
| Pt-dlp1 | LS991962 |
| Pt-dlp2 | LS991963 |
| Gm-dlp | LS991964 |
| Tc-dlp | LS991965 |
|  |  |
| Ek-sFRP125 | LS991966 |
| Pt-sFRP125 | LS991967 |
| Gm-sFRP125 | LS991968 |
|  |  |
| Ek-sFRP34 | LS991969 |
| Gm-sFRP34 | LS991970 |
|  |  |
| Ek-shf | LS991971 |
| Pt-shf | LS991972 |
| Gm-shf | LS991973 |
| Tc-shf | LS991974 |
