# Supplementary figures and images for: Gene expression analysis of potential morphogen signalling modifying factors in Panarthropoda
Source: EvoDevo. 2018 Sep 29;9:20. doi: 10.1186/s13227-018-0109-y (PMC6162966; doi:10.1186/s13227-018-0109-y)

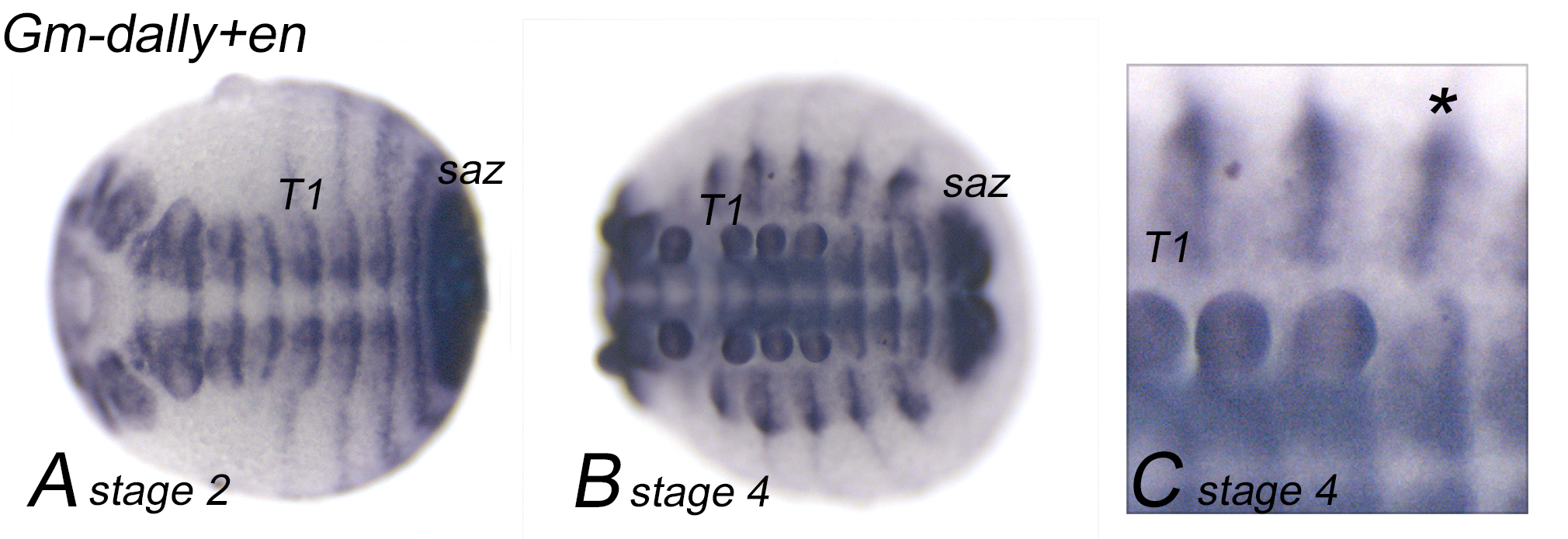

Supplement: Supplementary file 3 — Additional file 3: Fig. S1. Expression of Glomeris dally + engrailed (en). Abbreviations as in Fig. 2. Ventral views. The asterisk in c marks a single distinct stripe of enhanced expression in a dorsal segmental unit; this shows that en and dally are co-expressed (with no or minimal overlap of one gene’s expression compared to that of the other). Note that expression of en overlaps with the posterior of the dally-expressing domain in ventral segmental units. [file 13227_2018_109_MOESM3_ESM.tif]

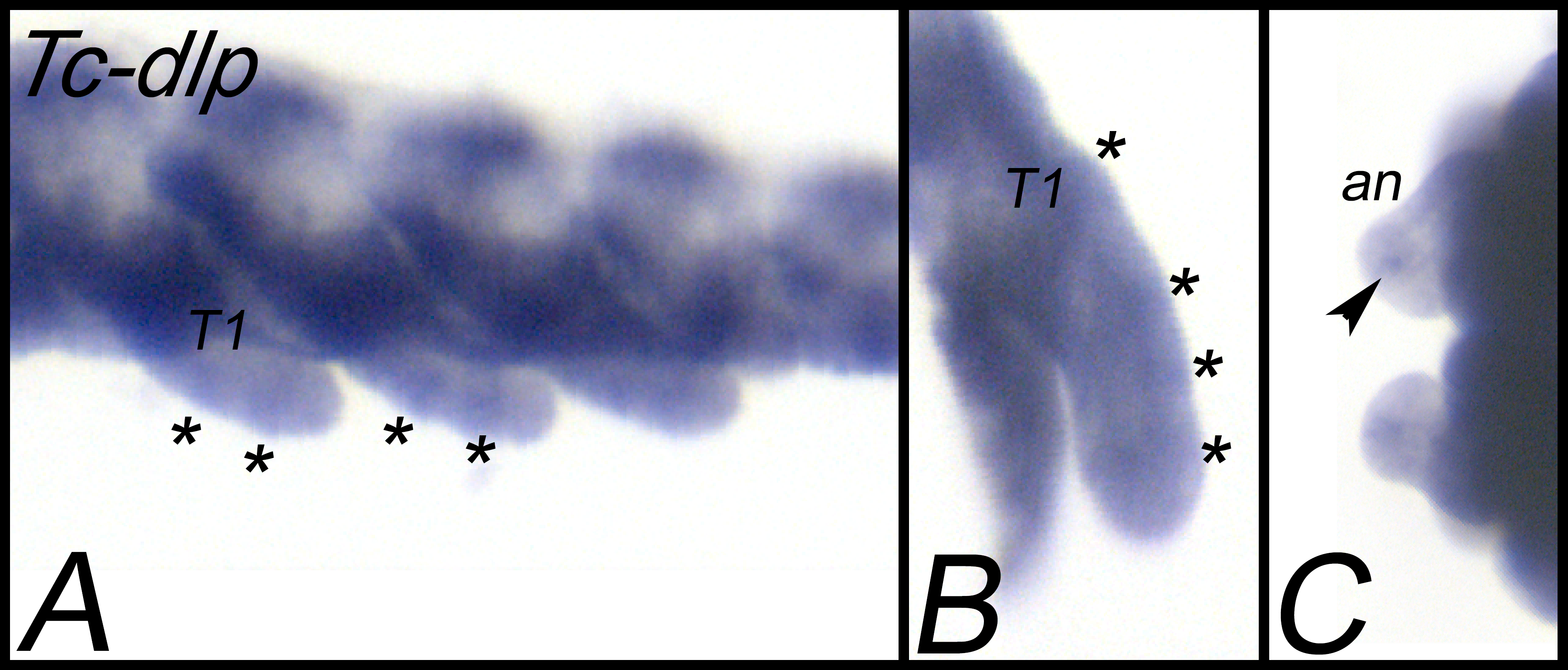

Supplement: Supplementary file 4 — Additional file 4: Fig. S2. Expression of Tribolium dlp in the legs and the antennae. Asterisks mark rings of expression in the legs. The arrowhead marks expression in the tip off the antenna. Abbreviations: an, antenna; T1, first thoracic leg. [file 13227_2018_109_MOESM4_ESM.tif]

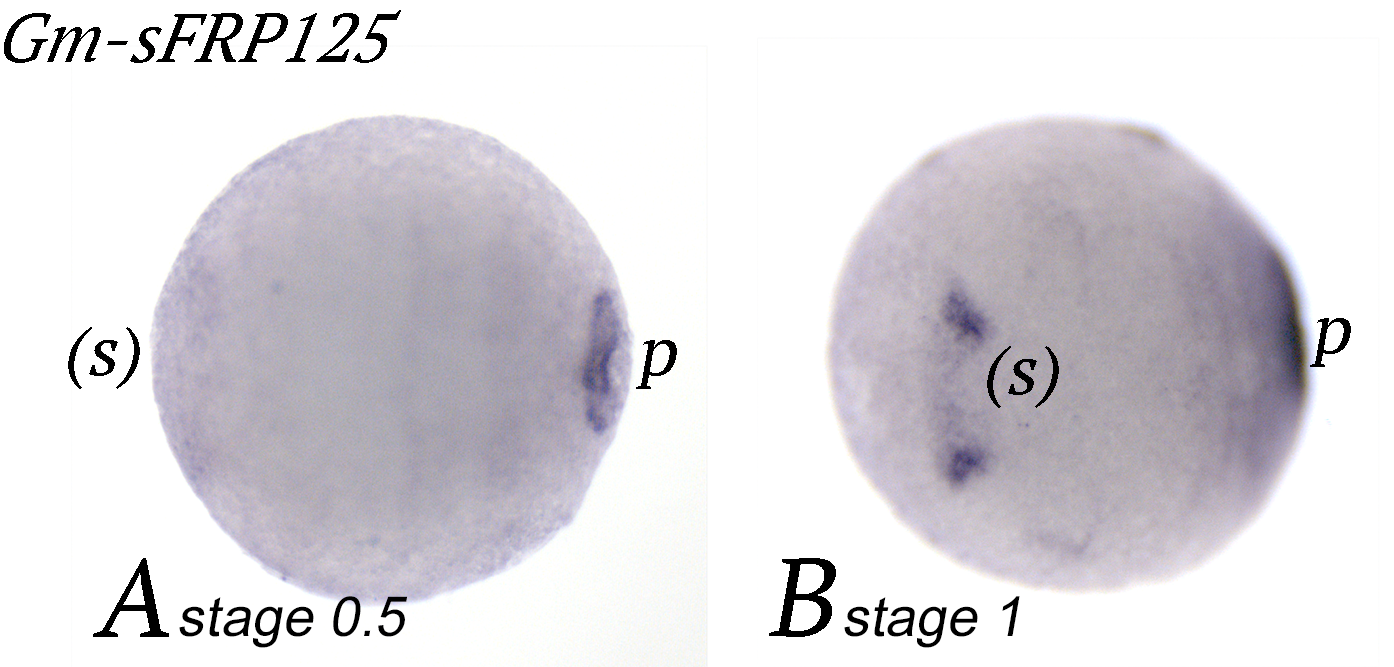

Supplement: Supplementary file 6 — Additional file 6: Fig. S4. Additional aspects of Glomeris sFRP125 expression. Abbreviations: p, proctodaeum; (s), primordium of the stomodaeum. See main body text for further information. [file 13227_2018_109_MOESM6_ESM.tif]

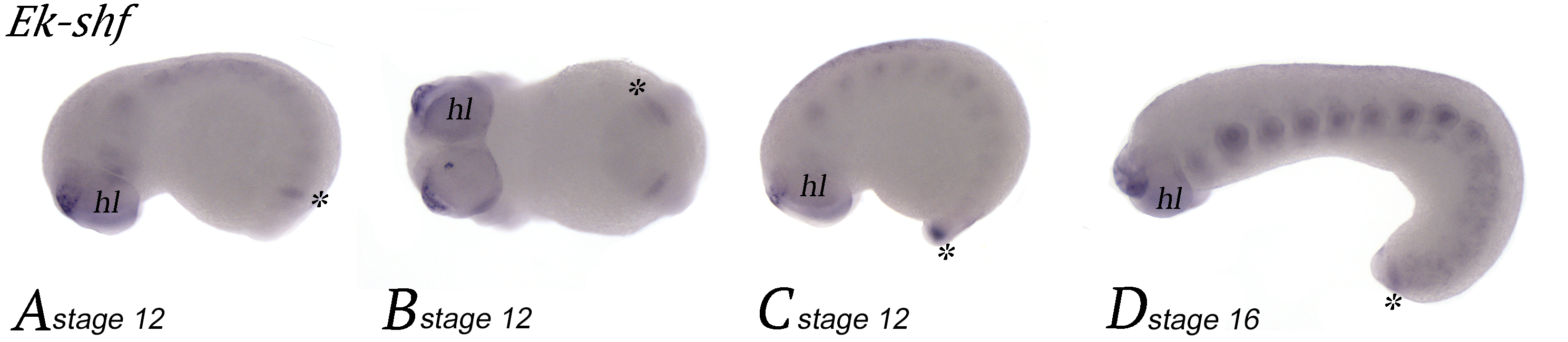

Supplement: Supplementary file 7 — Additional file 7: Fig. S5. Additional aspects of Euperipatoides shf expression. Abbreviations as in Fig. 2. Asterisks mark a stripe-like domain in the penultimate newly-formed segment (a, b, d) and in the posterior pit region (c). This expression was not seen in all embryos stained for shf and it may represent a dynamic and transient domain of expression, possibly involved in segment formation and/or patterning. [file 13227_2018_109_MOESM7_ESM.tif]
